# Neutralizing anti-IFN-γ IgG was increased in patients with systemic lupus erythematosus and associated with susceptibility to infection

**DOI:** 10.1007/s10067-023-06758-7

**Published:** 2023-10-19

**Authors:** Longfang Chen, Huihui Chi, Jialin Teng, Jianfen Meng, Hao Zhang, Yutong Su, Honglei Liu, Junna Ye, Hui Shi, Qiongyi Hu, ZhuoChao Zhou, Chengde Yang, Yue Sun, Xiaobing Cheng

**Affiliations:** Department of Rheumatology and Immunology, Ruijin Hospital, Shanghai Jiao Tong University School of Medicine, Shanghai, China

**Keywords:** Autoantibodies, Interferon-γ, Severe infections, Systemic lupus erythematosus

## Abstract

**Objectives:**

Systemic lupus erythematosus (SLE) is a complicated autoimmune disease, in which infection is a leading cause of death. Some SLE patients clinically presented with recurrent and refractory infections, which manifested as adult-onset immunodeficiency syndrome due to the production of anti-interferon-γ (anti-IFN-γ) autoantibodies. This study aimed to investigate the role of anti-IFN-γ autoantibodies concerning severe infections in SLE patients.

**Methods:**

We detected serum levels of anti-IFN-γ IgG/IgM isotypes in SLE patients with severe infections (*n* = 55), SLE patients without severe infections (*n* = 120), rheumatoid arthritis (*n* = 24), ankylosing spondylitis (*n* = 24), and healthy controls (*n* = 60). The relationship between anti-IFN-γ autoantibodies and clinical characteristics and laboratory parameters were analyzed. We further evaluated the neutralizing ability of anti-IFN-γ IgG.

**Results:**

The level of anti-IFN-γ IgG was significantly elevated in SLE patients with severe infections compared with the other groups (all *p* < 0.01), and the positive rates of anti-IFN-γ IgG in SLE patients with and without severe infections were 29.1% and 10.8%, respectively. Further analysis indicated that the levels of anti-IFN-γ IgG were positively associated with the SLEDAI score (r = 0.6420, *p* < 0.001), and it could predict the susceptibility to severe infections in SLE patients. Moreover, the inhibition and function assay showed that purified IgG from anti-IFN-γ IgG-positive SLE patients could neutralize IFN-γ, and further impair IFN-γ-induced STAT1 phosphorylation.

**Conclusions:**

The neutralizing anti-IFN-γ IgG might increase the susceptibility to infection in SLE patients, which has important implications for the treatment.

**Table:** 

**Key Points** *• The role of anti-IFN-γ autoantibodies concerning severe infections in SLE patients remains unknown.* *• The results of this study reveals that anti-IFN-γ IgG levels were significantly elevated in SLE patients with severe infections.* *• This study suggests that neutralizing anti-IFN-γ IgG might increase the susceptibility to infection in SLE patients.*

**Supplementary Information:**

The online version contains supplementary material available at 10.1007/s10067-023-06758-7.

## Introduction

Systemic lupus erythematosus (SLE) is a complicated systemic autoimmune disease typically characterized by the excessive production of autoantibodies due to abnormal recognition of self-antigens and the deposition of immune complexes in tissue [1]. According to a large multi-center cohort study of hospitalized patients with SLE in China, infection is still the leading cause of death which accounted for roughly 65.8% of deaths from SLE-related conditions [2]. Several variables, including disease-related factors, immunosuppressants, corticosteroid usage, and innate immunological dysregulations, are associated with the susceptibility to infection in SLE patients [3, 4]. Although the infectious disease is common, an underlying deficiency of the immune defense should be suspected when a patient experiences chronic, recurrent, or unusually severe infections with common pathogens [5].

Since 2004, cases of opportunistic infections related to anti-IFN-γ autoantibodies have been reported in adults, whose clinical presentation is similar to that of acquired immune deficiency syndrome [6–8]. Given that adults make up the majority of the disease's population, Browne et al. originally used the phrase "adult-onset immunodeficiency syndrome (AOID)" to define this unique form of illness [9].

The most prominent symptom in AOID patients with anti-IFN-γ autoantibodies is that the infection is difficult to control by standardized antimicrobial therapy [10]. Some SLE patients clinically presented with recurrent and refractory infections despite being treated with antibiotics aggressively, which is similar to those of AOID. It is still unclear whether anti-IFN-γ autoantibodies exist in patients with SLE and contribute to the susceptibility to infection. Thus, we detected serum anti-IFN-γ autoantibodies levels in a cohort of SLE patients with severe infections and compared them with those without severe infections and other diseases/healthy controls (HC). We also systematically investigated the profiles and clinical relevance of the anti-IFN-γ autoantibodies in SLE patients and further explored the neutralizing activity of the autoantibodies.

## Materials and methods

### Study population

We included 55 consecutive SLE patients with severe infections from the Department of Rheumatology and Immunology, Ruijin Hospital, Shanghai, China, from 2016 to 2021. Patients with cancer or diabetes were excluded. 120 SLE patients without severe infections who were matched for sex, age, basal glucocorticoid, and immunosuppressant usage in the last 6 months were included. All serological specimens from SLE patients were obtained when they were untreated during the first visit to our hospital. Besides, we randomly enrolled 24 rheumatoid arthritis (RA) patients and 24 ankylosing spondylitis (AS) patients with gender-age matched. SLE patients met the 2019 European League Against Rheumatism (EULAR) classification criteria [11, 12]. RA patients met the 2010 American College of Rheumatology (ACR)/EULAR classification criteria [13], and AS patients met the modified New York Criteria [14]. All patients received regular outpatient or telephone follow-ups. Demographic data, medical histories, clinical manifestations, and laboratory data such as anti-dsDNA antibody levels, positivity of anti-Sm antibody, anti-SSA antibody, anti-SSB antibody, anti-U1RNP antibody, anti-Ro52 antibody, and anti-nucleosome-A antibody, erythrocyte sedimentation rate (ESR), white blood cell counts (WBC), hemoglobin (Hb), platelets (PLT), C-reactive protein (CRP), complement 3 (C3), and complement 4 (C4) of SLE patients were collected. Disease activity was assessed by the SLE Disease Activity Index (SLEDAI) score [15]. Moreover, 60 gender-age matched HC without infectious, autoimmune, or autoinflammatory diseases were recruited. All serum samples were kept at -80 °C until use. Biological samples were obtained under a protocol approved by the Institutional Research Ethics Committee of Ruijin Hospital (2016–62), Shanghai, China, which was performed following the Declaration of Helsinki and the Principles of Good Clinical Practice. Informed consent was obtained from recruited subjects.

## Definition of severe infections

In our cohorts, severe infections were identified as requiring hospitalization or suffering from invasive complications, including bacterial infections (pneumonia, bacteremia, pyelonephritis, cellulitis, endocarditis, osteomyelitis, and septic arthritis), fungal infections (Candida albicans, aspergillosis, Cryptococcus neoformans, and pneumocystosis), viral infections (Epstein-Barr virus, herpes simplex virus, rubella virus, parainfluenza virus, and cytomegalovirus), and mycobacterial infections (tuberculosis and nontuberculous mycobacteria) [16]. All patients with severe infections received antimicrobial therapy. Specific details refer to the supplementary materials.

### Determination of autoantibodies against IFN-γ

Serum Anti-IFN-γ autoantibodies levels were determined by indirect enzyme-linked immunosorbent assay (ELISA) using a revised version of a method previously reported [17]. For details of the experiments, refer to the supplementary materials.

### Functional test for anti-IFN-γ autoantibodies

We purified total IgG from the serum samples and further evaluated the neutralizing ability of anti-IFN-γ IgG. The IFN-γ inhibition assay and functional assay for anti-IFN-γ IgG were performed, refering to the supplementary materials.

### Statistical analysis

Continuous variables were represented as median (interquartile range) or mean ± standard deviation (SD) according to distribution type, while categorical data were presented as frequency and percentages. For comparison between the two groups, the Mann–Whitney u-test was used for nonnormal distribution data, and the independent samples t-test was performed for normal distribution data. One-way ANOVA was used for the comparisons between multiple data sets. The correlations were evaluated by Pearson/Spearman correlation analysis according to distribution type. A two-sided *p*-value less than 0.05 was regarded as statistically significant. In this research, graphs were drawn using GraphPad Prism (version 8, GraphPad Software Inc., San Diego, USA), and data were analyzed using the SPSS software for Windows (version 26; SPSS Inc., Chicago, USA).

## Results

### Patient characteristics

We enrolled a total of 175 SLE patients, and the clinical data were represented in Table 1 and Table S1. The majority of the 55 SLE patients with severe infections presented with recurrent fever and often required a combination of drugs with different antimicrobial profiles. Baseline characteristics of RA patients, AS patients, and HC were shown in Table S2. All RA and AS patients rarely developed severe infections that required hospitalization. The study population consisted of 48 women and 7 men, and their median age was 32 years old. The SLE patients without severe infections group consisted of 104 women and 16 men, and the median age was 34 years old. Treatments were comparable between the two groups.

**Table 1:** Baseline characteristics of SLE patients with and without severe infections

Characteristic	SLE with severe infections (*n* = 55)	SLE without severe infections (*n* = 120)	*p* value
Age (mean ± SD, years)	39.4 ± 17.7	35.6 ± 11.6	0.530
Female (n, %)	48 (87.2)	104 (86.6)	0.829
SLEDAI (mean ± SD)	8.6 ± 5.1	5.3 ± 2.5	< 0.001***
Fever (n, %)	43 (78.2)	63 (52.5)	0.001**
Rash (n, %)	30 (54.5)	82 (68.3)	0.079
Arthritis (n, %)	31 (56.4)	60 (50.0)	0.378
Hematological involvement (n, %)	41 (74.5)	71 (59.1)	0.145
Lupus nephritis (proteinuria ≥ 0.5 g/24 h) (n, %)	34 (61.8)	58 (48.3)	0.098
Oral ulcer (n, %)	14 (25.5)	36 (30.0)	0.538
Alopecia (n, %)	8 (14.5)	26 (21.6)	0.270
Serositis (n, %)	9 (16.4)	18 (15.0)	0.817
Raynaud’s phenomenon (n, %)	6 (10.9)	11 (9.2)	0.719
Photosensitivity (n, %)	3 (5.5)	10 (8.3)	0.501
Vasculitis (n, %)	7 (12.7)	11 (9.2)	0.591
Neuropsychiatric manifestations (n, %)	5 (9.1)	5 (4.2)	0.089
Previous corticosteroid (n, %)	49 (89.1)	95 (79.2)	0.235
Daily prednisone dose (mean ± SD mg)	14.2 ± 11.9	12.6 ± 9.5	0.653
DMARDs use in the last 6 months (n, %)	21 (38.2)	41 (34.2)	0.533
ANA + (n, %)	55 (100)	120 (100)	1
Anti-dsDNA + (n, %)	35 (63.6)	78 (65.0)	0.154
Anti-Sm + (n, %)	20 (36.4)	38 (31.6)	0.541
Anti-SSA + (n, %)	32 (58.2)	68 (56.6)	0.851
Anti-SSB + (n, %)	13 (23.6)	25 (20.8)	0.677
Anti-U1RNP (n, %)	12 (21.8)	29 (24.2)	0.734
Anti-Ro 52 (n, %)	31 (56.4)	65 (54.2)	0.710
Anti-nucleosome-A + (n, %)	13 (23.6)	25 (20.8)	0.568

∗  ∗ *p* < 0.01, ∗  ∗  ∗ *p* < 0.001. *SLE*, systemic lupus erythematosus; *SD*, standard deviation; *SLEDAI*, SLE disease activity index; *DMARDs*, disease-modifying antirheumatic drugs

## Increased Serum levels of anti-interferon-γ autoantibodies in SLE patients with severe infections

**Fig. 1:**
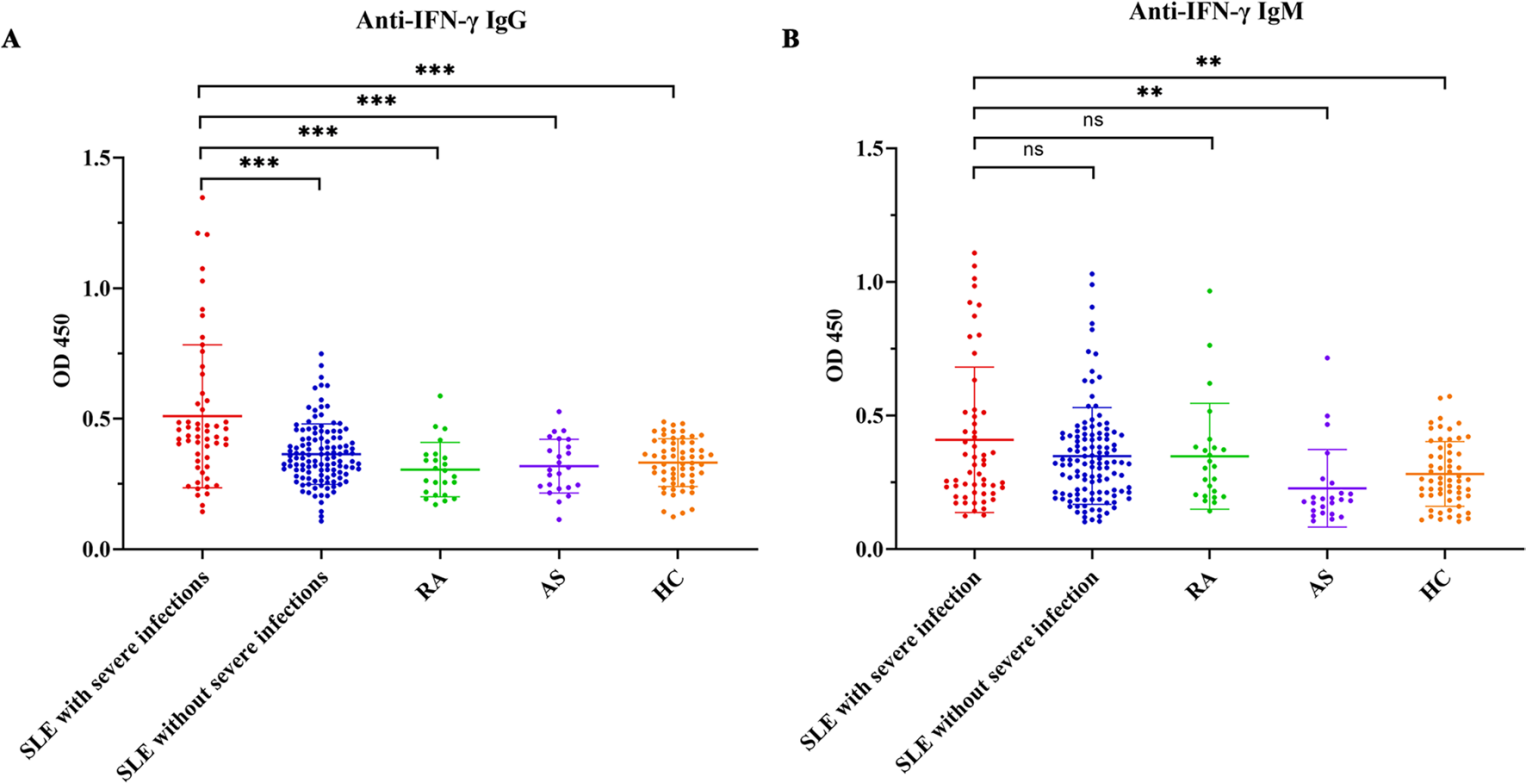
Elevated levels of anti-IFN-γ autoantibodies in SLE patients with severe infections. The serum levels of anti-IFN-γ IgG (**A**) and IgM (**B**) from SLE patients with severe infections (*n* = 55) or without severe infections (*n* = 120), patients with RA (*n* = 24), patients with AS (*n* = 24), and HC (*n* = 60). Values represent the means ± SD. **p < 0.01, and ***p < 0.001. *SLE*, systemic lupus erythematosus; *RA*, rheumatoid arthritis; *AS*, ankylosing spondylitis; *HC*, healthy controls

Elevated serum levels of anti-IFN-γ autoantibodies in AOID patients are the major cause of severe opportunistic infections, especially *Talaromyces marneffei* and *Nontuberculous mycobacteria* infections [9]. Similarly, we wondered whether the severe infections were also associated with anti-IFN-γ autoantibodies in SLE patients. First, we used an indirect ELISA to measure the serum levels of anti-IFN-γ IgG in patients with SLE, RA, AS, and HC. The results revealed that the anti-IFN-γ IgG levels were significantly higher in SLE patients with severe infections (0.51 ± 0.27) than those in SLE patients without severe infections (0.37 ± 0.09, *p* < 0.01), RA (0.30 ± 0.10, *p* < 0.001), AS (0.32 ± 0.10, *p* < 0.001), and HC (0.33 ± 0.09, *p* < 0.001) (Fig. 1A). No statistically significant differences were found between the SLE patients without severe infections group and RA, AS and HC group (all *p* > 0.05). For abnormal titers, we selected a threshold value of the mean plus 2 SD in the HC group, which is an OD value of 0.51. The positive rate of anti-IFN-γ IgG in SLE patients with severe infections, SLE patients without severe infections, AS, RA, and HC were 16/55 (29.1%), 11/120 (9.2%), 1/24 (4.2%), 1/24 (4.2%), and 0%, respectively, indicating that anti-IFN-γ IgG might be involved in the pathogenesis of SLE with severe infections.

In addition, we also detected anti-IFN-γ IgM using the same method, and no significant difference in serum anti-IFN-γ IgM levels between SLE patients with severe infections (0.41 ± 0.27) and those without severe infections (0.35 ± 0.18). As shown in Fig. 1B (*p* = 0.134). The levels of anti-IFN-γ IgM in SLE patients with severe infections were significantly higher than in AS (0.35 ± 0.19) and HC (0.28 ± 0.12) groups (all *p* < 0.01). However, there was no significant difference in IgM levels between the SLE patients without infection group and RA (0.23 ± 0.14), AS, and HC groups (*p* > 0.999, *p* = 0.051, and *p* = 0.178, respectively). These results revealed that the levels of anti-IFN-γ IgG, but not IgM, may play a potential pathogenic role in SLE patients with severe infections.

Furthermore, to explore the persistence of anti-IFN-γ autoantibodies, we measured the serum levels of anti-IFN-γ IgG/IgM from 5 anti-IFN-γ-positive SLE patients with severe infections at two different time points. The results were presented in Fig. S1. No significant difference was found in anti-IFN-γ IgG/IgM levels between the two groups, indicating that the anti-IFN-γ autoantibody may exist persistently in the serum.

### Distribution of infection subtypes at different anti-IFN-γ IgG levels in SLE patients with severe infections

**Fig. 2:**
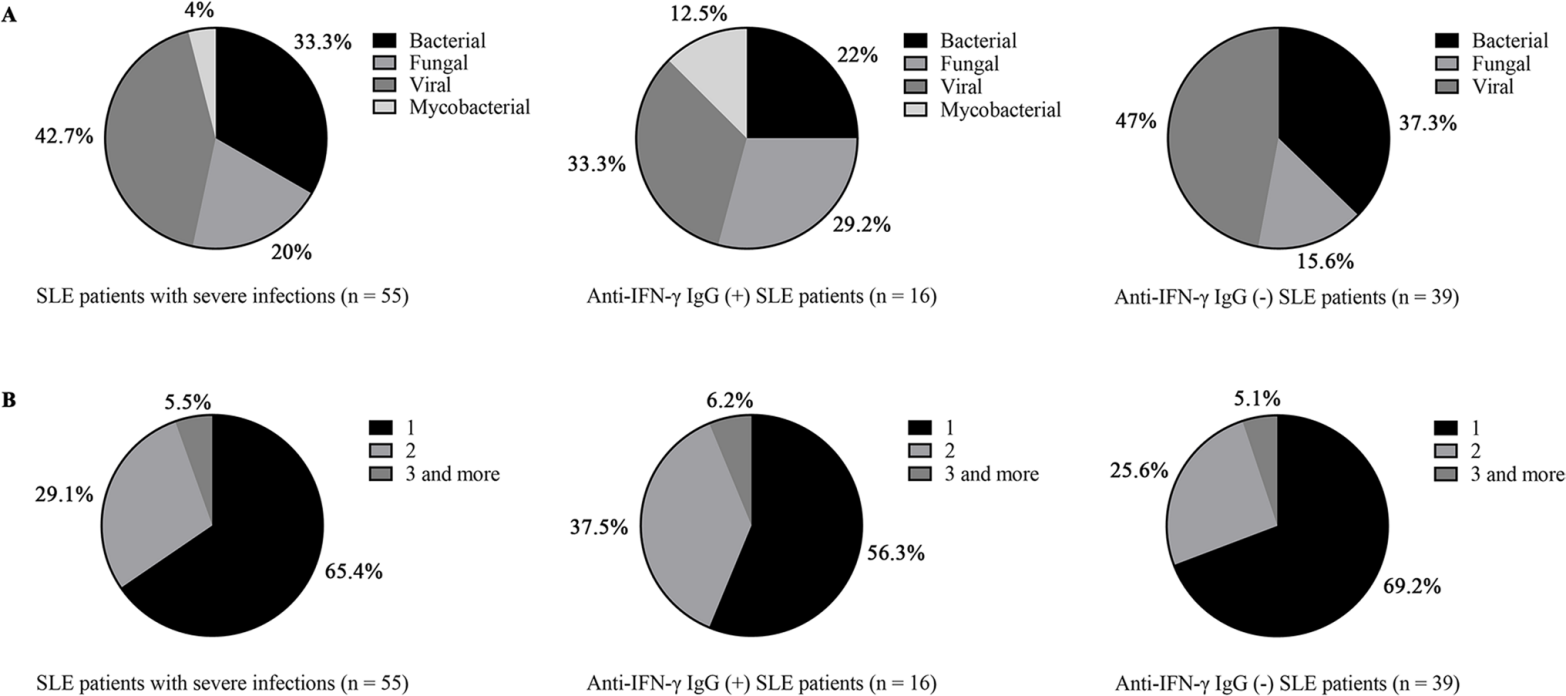
Distribution of infection subtypes and pathogenic species in SLE patients with severe infections. (**A**) Distribution of infection subtypes in SLE patients with severe infections. (**B**) Distribution of pathogenic species in SLE patients with severe infections. *SLE*, systemic lupus erythematosus

To gain further insight into the details of infections among SLE patients with severe infections, we analyzed the subtypes of pathogens among these patients. As illustrated in Fig. 2A, in SLE patients with severe infections, the percentage of bacterial, fungal, viral, and mycobacterial infections were 33.3%, 20%, 42.7%, and 4%, respectively. Among anti-IFN-γ IgG-positive SLE patients, the proportion of bacterial, fungal, viral, and mycobacterial infections were 22%, 29.2%, 33.3%, and 12.5%, respectively. In comparison, the composition ratio of bacterial, fungal, viral, and mycobacterial infections in anti-IFN-γ IgG-negative SLE patients were 37.3% (*p* = 0.448), 15.6% (*p* = 0.089), 47% (*p* = 0.369), and 0% (*p* = 0.006), respectively. A higher proportion of fungal and mycobacterial infections were observed in SLE patients with anti-IFN-γ IgG than those without anti-IFN-γ IgG. Figure 2B showed that in SLE patients with severe infections, 34.5% were co-infected with at least two pathogens. Among them, 43.7% of anti-IFN-γ IgG-positive SLE patients were infected with at least two pathogens, while 30.8% of anti-IFN-γ IgG-negative SLE patients were infected with at least two pathogens. The above data suggested that anti-IFN-γ IgG-positive SLE patients were more likely to develop fungal and mycobacterial infections than anti-IFN-γ IgG-negative SLE patients, and were also more likely to suffer from multiple infections.

## Correlation of anti-interferon-γ autoantibodies levels with disease activity

**Fig. 3:**
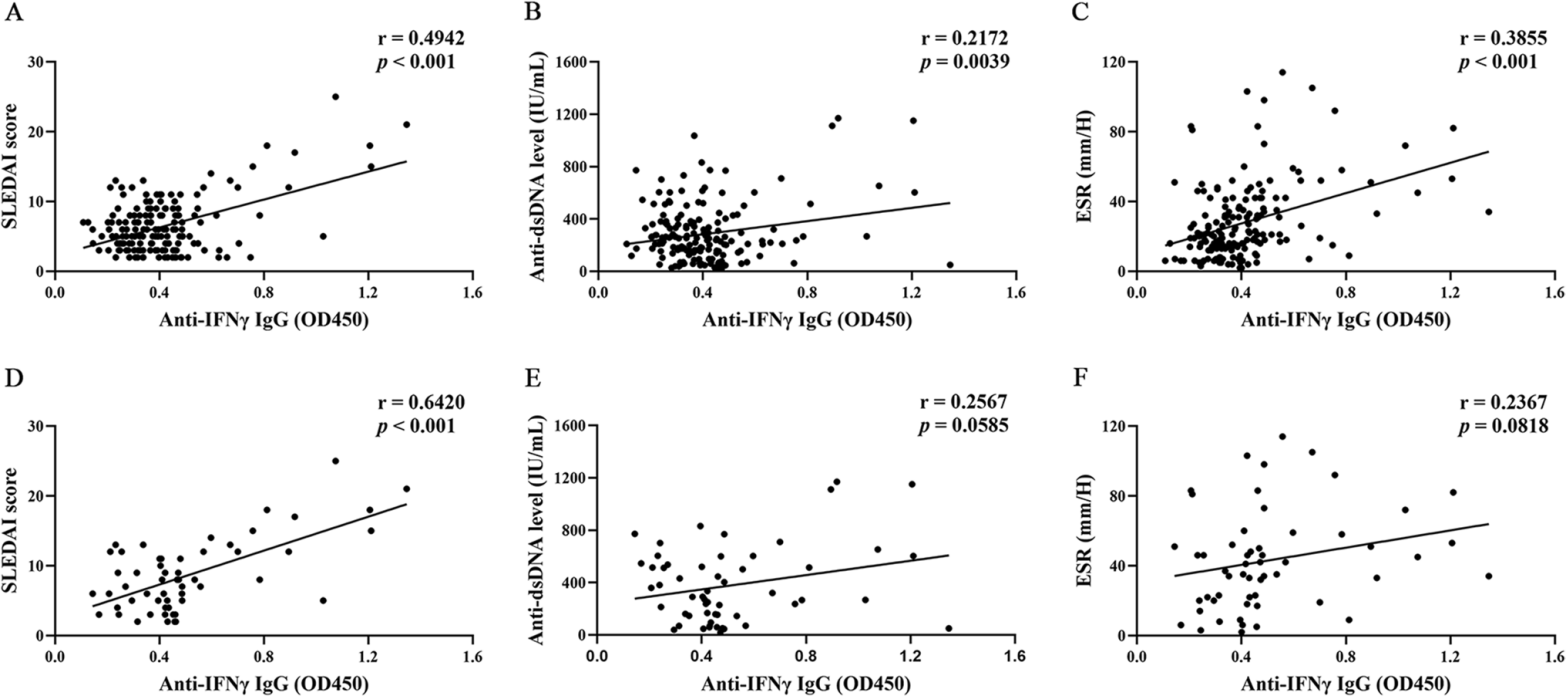
Anti-IFN-γ IgG levels positively correlated with SLEDAI score. The correlation between the levels of anti-IFN-γ IgG and SLE disease-related indicators including SLEDAI score (**A **and** D**), anti-dsDNA Ab (**B **and** E**), and ESR (**C **and** F**). **A **to** C** and **D **to** F** represent SLE patients (*n* = 170) and SLE patients with severe infections (*n* = 55), respectively. *SLE*, systemic lupus erythematosus; *SLEDAI*, SLE disease activity index; *ESR*, erythrocyte sedimentation rate; *Anti-dsDNA Ab*, anti-double-stranded DNA antibody; *ESR,* erythrocyte sedimentation rate.

Next, to evaluate the association between serum anti-IFN-γ autoantibodies levels and disease activity, we analyzed the correlation of anti-IFN-γ IgG levels with the SLEDAI score, anti-dsDNA antibody levels, and ESR in all SLE patients. Notably, the levels of anti-IFN-γ IgG were positively correlated with SLEDAI score (r = 0.4942, *p* < 0.001), anti-dsDNA antibody levels (r = 0.2172, *p* = 0.0039), and ESR (r = 0.3855,* p* < 0.001) (Fig. 3A-C), indicating that the anti-IFN-γ IgG levels might be associated with SLE disease activity. We also evaluated these correlations in SLE patients with severe infections. As illustrated in Fig. 3D-F, levels of anti-IFN-γ IgG were positively correlated with SLEDAI score (r = 0.6420, *p* < 0.001), but there was no significant association between anti-IFN-γ IgG levels and anti-dsDNA antibody levels or ESR levels (*p* = 0.0585, *p* = 0.818, respectively).

Besides, correlations of other laboratory data, such as WBC, Hb, PLT, CRP, C3, and C4, with anti-IFN-γ IgG levels were also investigated (all *p* > 0.05, Fig. S2 and S3). Furthermore, the correlations of anti-IFN-γ IgM levels with the above-mentioned laboratory indices were presented in Fig. S4 and S5. No significant correlations were found.

Finally, we analyzed the relationship between anti-IFN-γ autoantibodies with other autoantibodies in SLE patients with severe infections. As shown in Fig. S6, no significant difference was found between patients with seronegative and seropositive autoantibodies including anti-Sm, anti-SSA, anti-SSB, anti-U1RNP, anti-Ro52, and anti-nucleosome-A. The same goes for IgM.

## Association between anti-interferon-γ autoantibodies levels and the clinical manifestations in SLE patients

In addition to laboratory indicators, the correlation of anti-IFN-γ autoantibodies with the clinical parameters of SLE patients with severe infections was also investigated. As described in Table S3, higher levels of anti-IFN-γ IgG were observed in patients with fever and oral ulcers than in patients without fever and oral ulcers (*p* = 0.006, *p* = 0.004, respectively). No significant relationship was found between anti-IFN-γ IgG levels and other clinical manifestations, such as rash, arthritis, oral ulcer, alopecia, serositis, Raynaud’s phenomenon, photosensitivity, and vasculitis. Likewise, there are no significant correlations between anti-IFN-γ IgM levels and these clinical manifestations (Table S4).

## Receiver operating characteristic curves of anti-interferon-γ IgG in SLE patients with severe infections

**Fig. 4:**
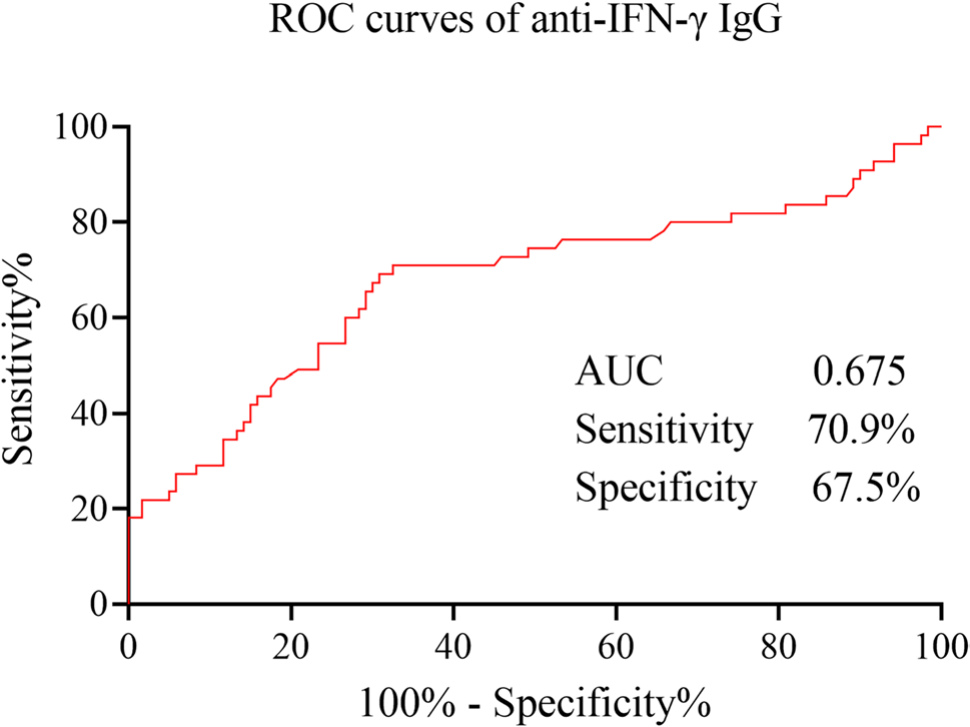
The ROC curves of anti-IFN-γ IgG in SLE patients with and without severe infections. *ROC*, receiver operating characteristic; *SLE*, systemic lupus erythematosus. *AUC,* areas under the curve

To investigate the probability of detecting anti-IFN-γ IgG for predicting severe infections in SLE patients, we calculated the receiver operating characteristic (ROC) curves (Fig. 4). The areas under the curve (AUC) of anti-IFN-γ IgG in SLE patients with and without severe infections were 0.675 (95% CI: 0.581–0.770, sensitivity, 70.9%; specificity, 67.5%: cut-off, 0.4; *p* = 0.0026). ROC curves demonstrated that the serum anti-IFN-γ IgG level might be a potentially useful biomarker to predict the risk of infections in SLE patients.

## Purified IgG from anti-IFN-γ IgG-positive SLE patients could neutralize IFN-γ.

**Fig. 5:**
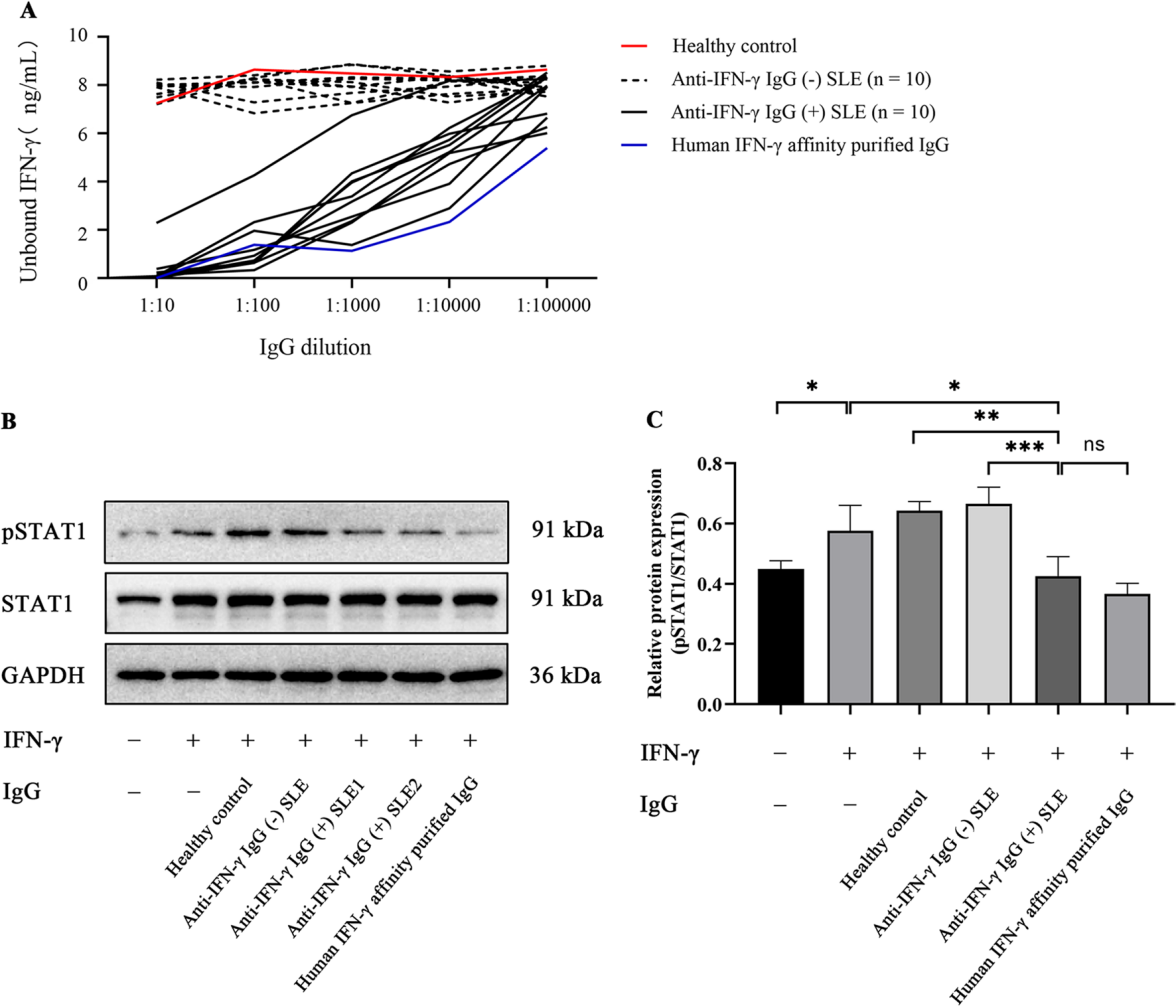
Purified IgG from anti-IFN-γ IgG-positive SLE patients neutralized IFN-γ. (**A**) Total IgG purified from anti-IFN-γ IgG-positive SLE patients interfered with the detection of human IFN-γ. Serially diluted IgG was respectively incubated with a fixed concentration (8 ng/ml) of IFN-γ. The amount of remaining unbound IFN-γ was detected by ELISA. (**B-C**) The phosphorylation of STAT1 (S727) was significantly decreased by anti-IFN-γ IgG in THP-1 cells. (**B**) Representative data for western blots. (**C**) Grayscale statistical map for western blots. Values were represented as means ± standard deviation (SD). *p < 0.05, **p < 0.01, ***p < 0.001. *SLE*, systemic lupus erythematosus; *ELISA*, enzyme-linked immunosorbent assay; *STAT1*: signal transducer and activator of transcription1. *pSTAT1*: phosphorylated-STAT1

Given the elevated levels of anti-interferon-γ autoantibodies might contribute to the pathogenesis of SLE with infections, we assessed the biological effects of these anti-IFN-γ IgG autoantibodies by inhibition assay and function assay. In the inhibition assay (Fig. 5A), a fixed concentration (8 ng/ml) of human recombinant IFN-γ was incubated with serially diluted IgG from the patients of SLE. IFN-γ was almost undetectable after incubation with total IgG from SLE patients defined as anti-IFN-γ IgG-positive, even with a high dilution rate (1:100,000 dilutions), suggesting a neutralizing ability of these autoantibodies against IFN-γ. We also carried out a function assay by evaluating whether the autoantibodies could reduce IFN-γ-induced phosphorylation of STAT1 in a human monocytic cell line THP-1. Firstly, we purified total IgG from 3 HC, 10 anti-IFN-γ IgG-negative SLE patients, and 10 anti-IFN-γ IgG-positive SLE patients (purity confirmed by Coomassie Brilliant Blue staining as shown in Fig. S6). As demonstrated in Fig. 5B and C, IFN-γ could remarkably upregulate the phosphorylation of STAT1, and IFN-γ-induced STAT1 phosphorylation was significantly inhibited after blocking with total IgG from anti-IFN-γ IgG-positive SLE patients. No inhibitory impact was seen with purified total IgG from healthy donors or the anti-IFN-γ IgG-negative SLE patients. Moreover, we collected IFN-γ levels on SLE patients with severe infections from the Department of Laboratory Medicine in our hospital. As shown in Fig. S8, all but one of the anti-IFN-γ-positive SLE patients had IFN-γ levels below the lower limit of detection (2.4 pg/ml). For the anti-IFN-γ-negative SLE group, three individuals had levels above the normal range, and one patient had a high IFN-γ level of 465 pg/ml. These data indicated that total IgG from anti-IFN-γ IgG-positive SLE patients can impair the function of IFN-γ.

## Discussion

In recent years, autoantibodies against one or more cytokines have been discovered in a variety of cases, including infections, cancer, and autoimmune illnesses, but little is understood about their clinical impact and spectrum [18, 19]. Anti-IFN-γ autoantibodies were first detected in individuals with severe *nontuberculous mycobacterial (NTM)* infections and were thought to be an autoimmune phenocopy of the interleukin-12 (IL-12)/IFN-γ axis inborn genetic abnormalities [20]. In addition, it has been shown that anti-IFN-γ autoantibodies underlie severe *Taralomyce marneffei* infection in HIV-negative patients [17]. Thus, anti-IFN-γ autoantibodies-associated AOID may be viewed as a novel kind of late-onset immunodeficiency that confer a propensity not only to severe *mycobacterial* but also to some fungal and bacterial infections. Here, we demonstrate that anti-IFN-γ IgG was significantly elevated in SLE patients with severe infections, and could be a potential biomarker to predict the risk of severe infections in SLE patients. Further functional experiments revealed that anti-IFN-γ IgG directly neutralized IFN-γ and inhibited IFN-γ-induced STAT1 phosphorylation, which might explain the susceptibility to infection in some SLE patients.

The excessive production of autoantibodies is a hallmark in the pathogenesis of SLE. Up to 27% of SLE patients have been shown to have autoantibodies against type I and type II interferons [21, 22]. Although it is unknown how they affect the immune responses and the pathogenesis of SLE, they have a significant potential to affect interferon signaling, disease activity, and responsiveness to biological treatments [22]. Gupta et al. analyzed the levels of 24 different anti-cytokine autoantibodies in 498 individuals with SLE, RA, and primary Sjogren's disease. In the end, anti-IFN-γ autoantibodies were nearly solely responsible for the elevated SLEDAI score and aberrant laboratory results in SLE patients [19]. This is consistent with our results. It is worth mentioning that the anti-IFN-γ IgG levels were positively correlated with other SLE diseases activity-related indicators, such as anti-dsDNA antibody levels and ESR levels, in all SLE patients, but the correlation was not obvious in SLE patients with severe infections. This indicated that there were no direct relationships between anti-IFN-γ IgG levels and disease activity, probably because infections could lead to disease exacerbation in SLE patients.

IFN-γ, a key immune system regulator which is important for preventing intracellular infections, is generated by natural killer (NK) cells, activated T cells, and macrophages [23]. After binding to its receptor, IFN-γ activates the JAK/STAT pathway and facilitates a variety of biological responses [24]. Anti-IFN-γ autoantibodies can block this binding, which inhibits the expression of STAT1 phosphorylation and the up-regulation of IL-12 and tumor necrosis factor (TNF)-α production [20, 25]. Additionally, individuals with anti-IFN-γ IgG might prevent IFN-γ-mediated antimicrobial immunity in macrophages and monocytes [26]. These involve IFN-γ-mediated polarization and M1 macrophage activation, the biosynthesis of reactive oxygen species (ROS), the secretion of chemokine, cytokine, and nitric oxide (NO)/iNO, and the effectiveness of phagocytosis and destruction [25, 27]. According to the epitope population, anti-IFN-γ autoantibodies neutralize IFN-γ signaling by blocking receptor binding or disrupting receptor assembly in several different ways [28]. Apart from the ability to block IFN-γ signaling, anti-IFN-γ autoantibodies may reduce IL-12p40 and CXCL-10 production by anchoring themselves to cells through IFN-γ/IFN-γR interactions, causing antibody-dependent cellular cytotoxicity to kill IFN-γR expressing cells via Fc-dependent activity [28]. Overall, these molecular characteristics and functional analyses of anti-IFN-γ autoantibodies provided significant insights into the production of these autoantibodies and the underlying mechanisms by which they compromise host immunity, thus explaining their correlation with susceptibility to various infections.

As mentioned before, *NTM* infection is the most common infectious disease in AOID [29]. However, other opportunistic pathogens such as *Salmonella* species, *Burkholderia pseudomallei*, *Histoplasma capsulatum*, *Cryptococcus neoformans*, and *Varicella-zoster virus* are also frequently seen in this patient population [9, 30, 31]. More precise indicators of the immunocompromised status in these individuals include concurrent infections with at least 2 opportunistic pathogens [9]. In our anti-IFN-γ IgG-positive SLE cohort, viral and fungal infections were more common, together accounting for 62.5% of the cases. Moreover, the proportion of mycobacterial infections was also significantly increased compared with the anti-IFN-γ IgG-negative SLE patients, predicting the mechanism of IFN-γ-mediated resistance to intracellular bacterial impairment. Similarly, about half of them had 2 or more co-infections at the same time, which means they are more likely to suffer from multiple infections.

For patients with AIOD, standardized antimicrobial treatment is by far the most effective therapy, but antibiotic therapy alone still has difficulty controlling the infection. Browne et al. used rituximab to treat 4 AOID patients with high titers of anti-IFN-γ autoantibodies and invasive *NTM* who were progressing despite aggressive antimicrobial therapy. Following treatment with different doses of rituximab, clinical remission was achieved in all patients, with reduced antibody titers and recovery of IFN-γ pathway responses [32]. Paradoxically, the most common advert event of rituximab is infection. How to balance infection with the use of immunosuppressants is a difficult issue. On the one hand, antibody production needs to be suppressed, and on the other hand, the clearance of B cells causes a decrease in the patient's ability to defend against pathogens. It is worth mentioning that in our anti-IFN-γ antibodies-positive SLE cohort, two patients were treated with rituximab after the infection was controlled, and after multiple rituximab consolidation treatments, both two patients were subsequently stable, with no recurrent severe infections and no frequent lupus activity. We believe that patients who are positive for anti-IFN-γ IgG have an increased susceptibility to severe infections and need to be closely monitored for infection-related indicators during follow-up. If patients with high titers of anti-IFN-γ autoantibodies presented with recurrent and refractory infections, more aggressive treatments such as plasmapheresis to deplete monoclonal antibodies, B-cell depletion, and suppression of B-cell survival factors could be taken into consideration [32–34].

Our study, for the first time, revealed that the presence of anti-IFN-γ autoantibodies in patients with SLE indicated a high risk of developing severe infections. Additionally, this finding paves the way for therapeutic interventions in SLE patients with autoantibodies against IFN-γ and severe infections. However, the limitations of this study should be acknowledged. This was an exploratory study with a relatively small sample size conducted in one medical center, which may cause selection bias. Second, as our study was a retrospective design, the levels of IFN-γ and its autoantibodies during infection were not detected. Third, we only have 5 serial serum samples of anti-IFN-γ IgG positive SLE patients, which may not enough to extrapolate to all anti-IFN-γ IgG positive SLE patients. Prospective multi-center studies with larger sample sizes were needed to validate these results and to explore the role of discriminating SLE patients with severe infections from those without severe infections.

## Conclusions

Our study firstly reports that at least 29% of SLE patients with severe infections have autoantibodies against IFN-γ. The elevated levels of anti-IFN-γ IgG were associated with SLEDAI score, and the ROC curves illustrated that anti-IFN-γ IgG levels could predict the susceptibility of SLE patients to severe infections, indicating that anti-IFN-γ IgG is a novel disease biomarker. In addition, our findings demonstrate that purified IgG from anti-IFN-γ IgG-positive SLE patients could neutralize IFN-γ, and further impair IFN-γ-induced STAT1 phosphorylation, which is related to the susceptibility and severity of infection in SLE.

### Supplementary Information

Below is the link to the electronic supplementary material.

Supplementary file1 (DOCX 1217 KB)

Supplementary file2 (PZFX 27 KB)

Supplementary file3 (DOCX 1359 KB)

## Data Availability

The datasets generated during and/or analyzed during the current study are available from the corresponding author on reasonable request.
