# Supplementary material for: Neutralizing anti-IFN-γ IgG was increased in patients with systemic lupus erythematosus and associated with susceptibility to infection
Source: Clin Rheumatol. 2023 Oct 19;43(1):189–98. doi: 10.1007/s10067-023-06758-7 (PMC10774216; doi:10.1007/s10067-023-06758-7)
Supplement: Supplementary file 1 — Supplementary file1 (DOCX 1217 KB) [file 10067_2023_6758_MOESM1_ESM.docx]

**Supplementary materials and methods**

**Determination of autoantibodies against IFN-γ**

In brief, recombinant human IFN-γ (2 μg/ml, Beyotime, China) were added 100 μl/well to a 96-well high-binding plate (Corning, New York, USA), and incubated overnight at 4°C. Then, the wells were washed with PBST buffer (PBS plus 0.05% Tween-20) and blocked with PBST buffer containing 5% bovine serum albumin (BSA, Biofroxx, Germany) for 2 h at room temperature (RT). Next, 100ul serum samples (1:100 diluted with blocking buffer) were added to per well and incubated for 2 h at RT after the plates were washed. Then, plate washing was operated, and 100 μl of horseradish peroxidase (HRP)-conjugated goat anti-human IgG (1:5,000 diluted; Abcam, UK) or HRP-conjugated goat anti-human IgM (1:5,000 diluted; Abcam, UK) were added to each well for another 1 h at RT. After that, the plate was washed, and 100 μl of tetramethylbenzidine substrate solution was added to per well. It was then stopped by the addition of 100 μl of 0.5 M H2SO4 and determine the optical density (OD) of each well at 450 nm in a microplate reader (Bio-Rad Laboratories, Richmond, USA).

**Purification and identification of IgG**

Total IgG was obtained from the serum samples by a Protein G Agarose Pre-loaded column (Fast Flow, 1ml, Beyotime, China) according to the manufacturer’s instructions and concentrated in a centrifuge tube (100kDa, Millipore, Germany). Furthermore, the isolated IgG was confirmed by Coomassie Brilliant Blue staining (Beyotime, China).

**IFN-γ inhibition assay**

Total IgG isolated from SLE patients and HC was incubated with recombinant human IFN-γ at a terminal concentration of 8 ng/ml for 3 h at RT after serial dilutions (1:10; 1:100; 1:1,000; 1:10,000, and 1:100,000). The levels of remaining unbound IFN-γ levels were detected with a human IFN-γ ELISA kit (Cusabio, China) following the manufacturer’s instructions.

**Functional assay for anti-IFN-γ IgG**

We evaluated the neutralizing activity of anti-IFN-γ IgG by assessing their ability to decrease the IFN-γ-induced Phosphorylated-Signal Transducer and Activator of Transcription1 (pSTAT1) upregulation on THP-1 cells. THP-1 cells were incubated in a complete RPMI-1640 medium with 10% FBS (Gibco, USA), 1% L-Glutamine (Thermofisher, USA), and 1% penicillin/streptomycin (Gibco, USA). THP-1 cells (1×10^6 cells/ml) were cultured with a fresh medium containing human recombinant IFN-γ (20 ng/ml, Beyotime, China), in the absence or presence of purified human immunoglobulin G (200 ug/ml), total IgG from serum samples or Human IFN-γ affinity-purified Ab (10ug/ml) for 15 min at 37 °C. After washing with PBS, total protein was extracted from the THP-1 cells for detecting the expression of pSTAT1 by western blotting.

**Western blotting**

The THP-1 cells were digested in RIPA buffer containing phosphatase inhibitor cocktail (Yeasen Biotechnology Co., Ltd), and protease inhibitor Cocktail (Yeasen Biotechnology Co., Ltd). Then, we separated 20 μg of proteins using 10% SDS polyacrylamide gels and subsequently transferred them onto a polyvinylidene difluoride (PVDF) membrane (Millipore, US). The membrane was incubated in protein-free rapid blocking buffer (Epizyme, China) for 10 min at RT and then cultured with the related primary antibodies: anti-STAT1 antibody (Cusabio, China); Anti-phospho-STAT1 antibody (Abcam, UK) and anti-GAPDH antibody (Servisebio, China) overnight at 4 ˚C on shaking table. Next, incubation with secondary anti-rabbit HRP (1:5,000 diluted, Cell Signal Technology, US) was performed at RT for 1 h. Finally, we used an Electrochemiluminescence (ECL) Plus Western Blot Detection Kit (Invitrogen, Thermo Fisher Scientific) for protein quantification and ImageJ V1.48(National Institutes of Health) for analysis.

**Table S1. Specific characteristics of the 55 SLE patients with severe infections.**

| ID | Age | Sex | Daily prednisone dose | DMARDs in the last 6 months | Pathogens | Anti-IFN-γ IgG |
| --- | --- | --- | --- | --- | --- | --- |
| 1 | 18 | F | 0 | / | *C. albicans* | Negative |
| 2 | 59 | F | 15 | / | *P. aeruginosa* | Negative |
| 3 | 51 | M | 30 | CTX | TB | Positive |
| 4 | 29 | F | 10 | CTX, Tacrolimus | EBV | Negative |
| 5 | 22 | F | 15 | CTX, MMF | HSV | Negative |
| 6 | 18 | F | 10 | MMF | RV, HSV | Negative |
| 7 | 30 | F | 5 | MTX | RV | Negative |
| 8 | 64 | F | 0 | / | *S. aureus*, EBV | Negative |
| 9 | 85 | M | 7.5 | Cyclosporin A | *Salmonella enteritidis*, PFV | Positive |
| 10 | 58 | F | 10 | CTX | *C. albicans*, *S. maltophilia* | Negative |
| 11 | 56 | F | 50 | / | HSV | Negative |
| 12 | 24 | F | 0 | / | EBV | Negative |
| 13 | 32 | F | 0 | / | EBV | Negative |
| 14 | 82 | F | 0 | / | HSV | Negative |
| 15 | 43 | F | 0 | / | *K. pneumoniae*, CMV | Negative |
| 16 | 28 | F | 0 | / | *E.coli*, CMV, EBV | Negative |
| 17 | 31 | M | 30 | CTX | *S. aureus*, CMV | Positive |
| 18 | 53 | F | 50 | / | *S. pneumoniae*, EBV | Negative |
| 19 | 29 | F | 35 | CTX, Tacrolimus, MMF | Aspergillus, TB | Positive |
| 20 | 22 | F | 5 | / | *C. albicans*, HSV, EBV | Positive |
| 21 | 28 | F | 30 | Thalidomide | *C. albicans*, HSV | Negative |
| 22 | 32 | F | 5 | / | *E.coli* | Negative |
| 23 | 32 | F | 0 | / | EBV | Negative |
| 24 | 36 | F | 0 | / | HSV | Negative |
| 25 | 30 | F | 30 | / | HSV | Negative |
| 26 | 40 | F | 0 | / | *C. neoformans* | Positive |
| 27 | 21 | F | 30 | / | *Aspergillus* | Positive |
| 28 | 36 | F | 0 | / | *S. aureus* | Negative |
| 29 | 23 | F | 0 | Zazthioprine | *S. maltophilia* | Negative |
| 30 | 24 | F | 10 | MMF | *E.coli* | Negative |
| 31 | 26 | F | 15 | CTX | *K. pneumoniae* | Positive |
| 32 | 72 | F | 30 | / | *C. albicans* | Negative |
| 33 | 26 | F | 40 | / | *S. aureus* | Negative |
| 34 | 60 | F | 10 | / | EBV | Negative |
| 35 | 41 | F | 10 | Cyclosporin A | *P. aeruginosa, S. pneumoniae* | Negative |
| 36 | 29 | F | 15 | Tacrolimus | CMV | Positive |
| 37 | 40 | F | 20 | CTX, MMF | *E.coli*, CMV | Negative |
| 38 | 20 | F | 10 | / | *Aspergillus*, HSV | Positive |
| 39 | 34 | M | 0 | / | *S. pneumoniae* | Negative |
| 40 | 16 | F | 0 | / | *C. albicans, E.coli* | Positive |
| 41 | 32 | F | 20 | / | *P. aeruginosa* | Positive |
| 42 | 18 | F | 20 | / | CMV | Negative |
| 43 | 20 | F | 0 | / | EBV | Positive |
| 44 | 74 | F | 30 | / | *S. pneumoniae* | Positive |
| 45 | 53 | F | 50 | CTX | CMV, TB | Positive |
| 46 | 27 | F | 7.5 | CTX | *S. maltophilia* | Negative |
| 47 | 44 | F | 10 | / | EBV | Positive |
| 48 | 58 | F | 10 | / | *Aspergillus*, EBV | Negative |
| 49 | 34 | M | 15 | CTX, MMF | *P. aeruginosa* | Negative |
| 50 | 34 | M | 7.5 | / | *Aspergillus* | Negative |
| 51 | 63 | F | 0 | / | EBV | Negative |
| 52 | 53 | M | 0 | / | CMV | Negative |
| 53 | 65 | F | 30 | / | *C. albicans, E.coli*, HSV | Negative |
| 54 | 62 | F | 40 | CTX, MMF | *Salmonella enteritidis*, HSV | Negative |
| 55 | 28 | F | 15 | CTX | *P. aeruginosa*, RV | Negative |

*DMARDs*, disease-modifying antirheumatic drugs; *CTX*, [Cyclophosphamide](javascript:;); *MMF*, [Mycophenolate](javascript:;) [Mofetil](javascript:;); *C. albicans*, Candida albicans; *P. aeruginosa,* Pseudomonas aeruginosa; *TB*, [mycobacterium](javascript:;) [tubercualosis](javascript:;); *EBV*, [Epstein-Barr](javascript:;) [virus](javascript:;); *HSV*, [herpes](javascript:;) [simplex](javascript:;) [virus](javascript:;); *RV*, [rubella](javascript:;) [virus](javascript:;); *S. aureus*, Staphylococcus aureus; *PFV*, [parainfluenza](javascript:;) [virus](javascript:;); *S. maltophilia*, Stenotrophomonas maltophilia; *C. neoformans*, Cryptococcus neoformans; *CMV*, [cytomegalovirus](javascript:;); *K. pneumoniae*, Klebsiella pneumoniae.

**Table S2. Baseline characteristics of RA patients, AS patients and HC.**

| Characteristic | RA (n = 24) | AS (n = 24) | HC (n = 60) |
| --- | --- | --- | --- |
| Age (mean ± SD, years) | 38.1 ± 15.6 | 39.0 ± 15.2 | 37.5 ± 15.6 |
| Female (n, %) | 21 (87.5) | 21 (87.5) | 52 (86.7) |
| Daily prednisone dose (mean ± SD mg) | 10.2 ± 1.25 | 0.83 ± 0.47 | / |
| DMARDs use in the last 6 months (n, %) | (17, 70.8%) | (23, 95.8) | / |

*RA*, rheumatoid arthritis; *AS*, ankylosing spondylitis; *HC*, healthy controls. *DMARDs*, disease-modifying antirheumatic drugs.

**Table S3. Comparison of anti-IFN-γ IgG according to disease manifestations in SLE patients with severe infections.**

|  | Anti-IFN-γ IgG (OD450) | | *p* value |
| --- | --- | --- | --- |
| Fever | (+), n = 43 | 0.462, 0.327 | 0.006** |
|  | (-), n = 12 | 0.412, 0.138 |  |
| Rash | (+), n = 30 | 0.461, 0.339 | 0.148 |
|  | (-), n = 25 | 0.423, 0.170 |  |
| Arthritis | (+), n = 31 | 0.423, 0.204 | 0.387 |
|  | (-), n = 24 | 0.467, 0.207 |  |
| Oral ulcer | (+), n = 14 | 0.552, 0.374 | 0.004** |
|  | (-), n = 41 | 0.422, 0.161 |  |
| Alopecia | (+), n = 8 | 0.470, 0.214 | 0.197 |
|  | (-), n = 47 | 0.430, 0.249 |  |
| Serositis | (+), n = 7 | 0.487, 0.333 | 0.633 |
|  | (-), n = 46 | 0.433, 0.168 |  |
| Raynaud’s phenomenon | (+), n = 6 | 0.442, 0.214 | 0.777 |
|  | (-), n = 49 | 0.435, 0.219 |  |
| Photosensitivity | (+), n = 3 | 0.404, 0.107 | 0.394 |
|  | (-), n = 52 | 0.444, 0.227 |  |
| Vasculitis | (+), n = 7 | 0.401, 0.120 | 0.143 |
|  | (-), n = 48 | 0.456, 0.254 |  |

Anti- IFN-γ IgG (OD 450) was shown as median, interquartile range, and differences between the two groups were analyzed using the Mann-Whitney *U* test. ∗∗*p* < 0.01. *SLE*, systemic lupus erythematosus.

**Table S4. Comparison of anti-IFN-γ IgM according to disease manifestations in SLE patients with severe infections.**

|  | Anti-IFN-γ IgM (OD450) | | *p* value |
| --- | --- | --- | --- |
| Fever | (+), n = 43 | 0.255, 0.304 | 0.058 |
|  | (-), n = 12 | 0.429, 0.123 |  |
| Rash | (+), n = 30 | 0.244, 0.280 | 0.056 |
|  | (-), n = 25 | 0.385, 0.215 |  |
| Arthritis | (+), n = 31 | 0.316, 0.233 | 0.964 |
|  | (-), n = 24 | 0.303, 0.320 |  |
| Oral ulcer | (+), n = 14 | 0.326, 0.295 | 0.643 |
|  | (-), n = 41 | 0.288, 0.238 |  |
| Alopecia | (+), n = 8 | 0.249, 0.073 | 0.283 |
|  | (-), n = 47 | 0.345, 0.290 |  |
| Serositis | (+), n = 7 | 0.345, 0.283 | 0.617 |
|  | (-), n = 46 | 0.285, 0.280 |  |
| Raynaud’s phenomenon | (+), n = 6 | 0.245, 0.113 | 0.508 |
|  | (-), n = 49 | 0.317, 0.281 |  |
| Photosensitivity | (+), n = 3 | 0.802, 0.466 | 0.317 |
|  | (-), n = 52 | 0.302, 0.250 |  |
| Vasculitis | (+), n = 7 | 0.345, 0.181 | 0.256 |
|  | (-), n = 48 | 0.272, 0.292 |  |

Anti- IFN-γ IgM (OD 450) was shown as median, interquartile range, and differences between the two groups were analyzed using the Mann-Whitney *U* test. No significant difference was found. *SLE*, systemic lupus erythematosus.

**Supplementary figures**


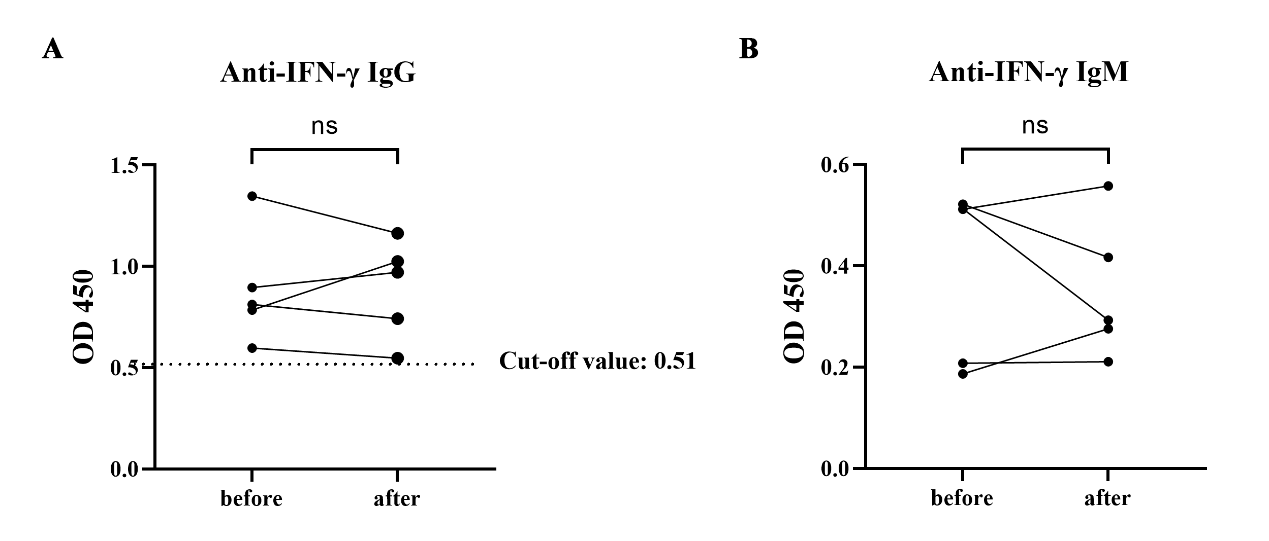
 **Figure S1. Serial levels of anti-IFN-γ autoantibodies in anti-IFN-γ-positive SLE patients.** The serum levels of anti-IFN-γ IgG (A) and IgM (B) from anti-IFN-γ-positive SLE patients with severe infections (n = 5) at two different time points. Values represent the means ± SD. The positive cut-off value is 0.51. *SLE*, systemic lupus erythematosus.

**
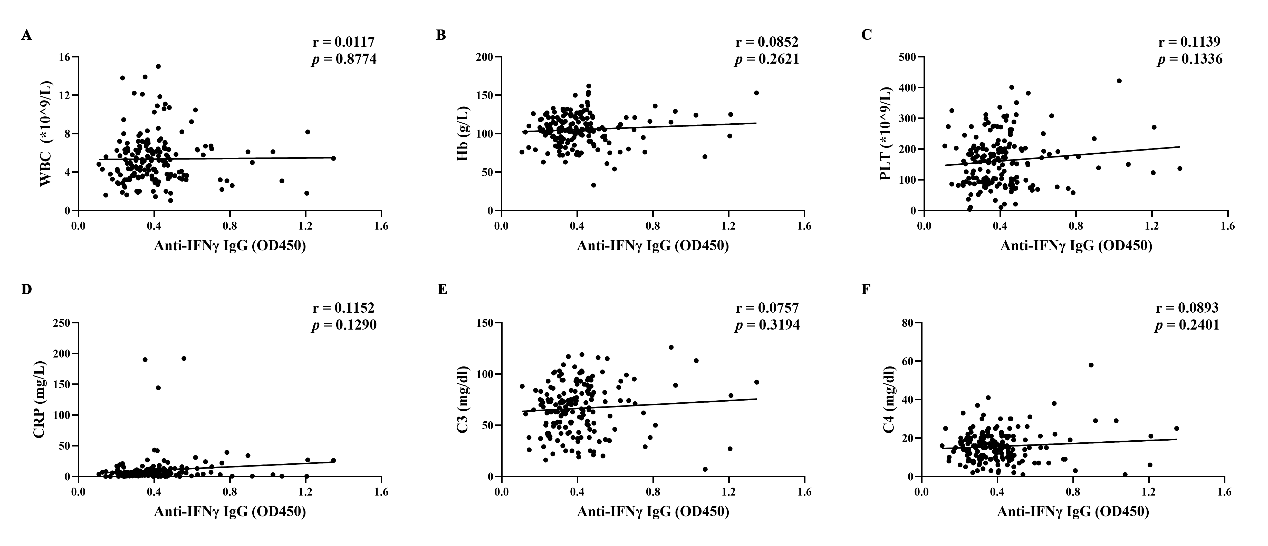
**

**Figure S2. The correlation of anti-IFN-γ IgG levels and other laboratory data in patients with SLE.** The correlation between anti-IFN-γ IgG levels and WBC (A), Hb (B), PLT (C), CRP (D), C3 (E), and C4 (F) in SLE patients (n = 175). No significant difference was found. *SLE*. systemic lupus erythematosus; *WBC*. white blood cells; *Hb*, haemoglobin; *PLT*, platelets; *CRP*, C-reactive protein; *C3*, complement 3; *C4*, complement 4.


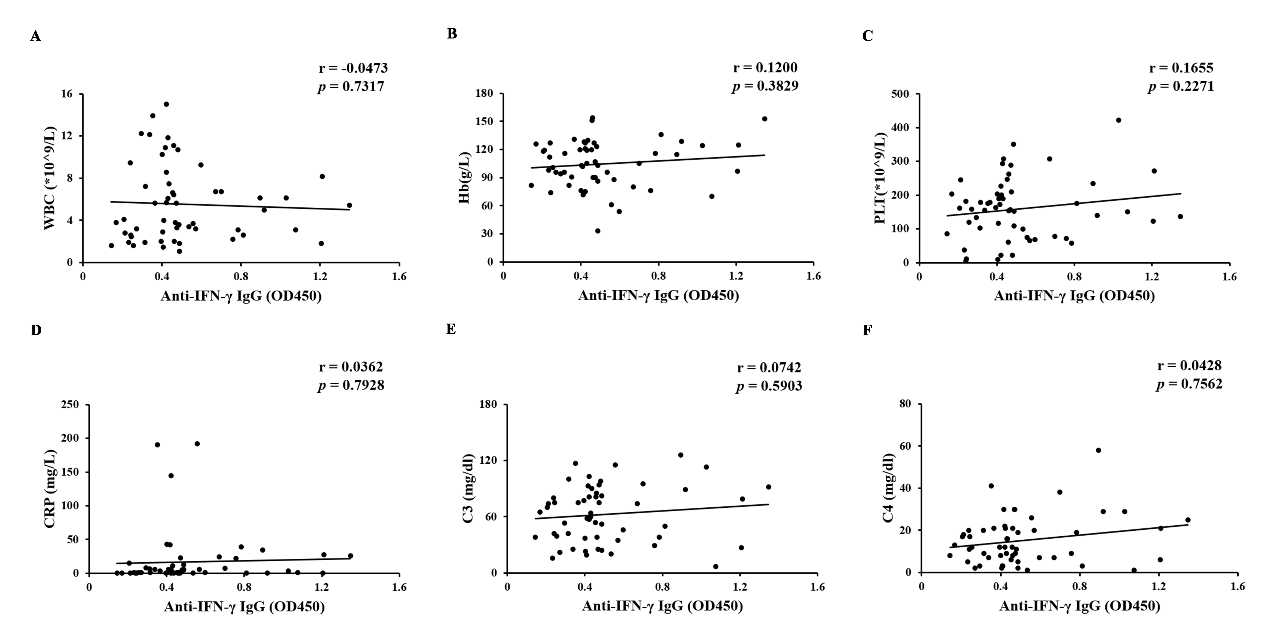


**Figure S3. The correlation of anti-IFN-γ IgG levels and other laboratory data in SLE patients with severe infections.** The correlation between anti-IFN-γ IgG levels and WBC (A), Hb (B), PLT (C), CRP (D), C3 (E), and C4 (F) in SLE patients with severe infections (n = 55). No significant difference was found. *SLE*. systemic lupus erythematosus; *WBC*. white blood cells; *Hb*, haemoglobin; *PLT*, platelets; *CRP*, C-reactive protein; *C3*, complement 3; *C4*, complement 4.


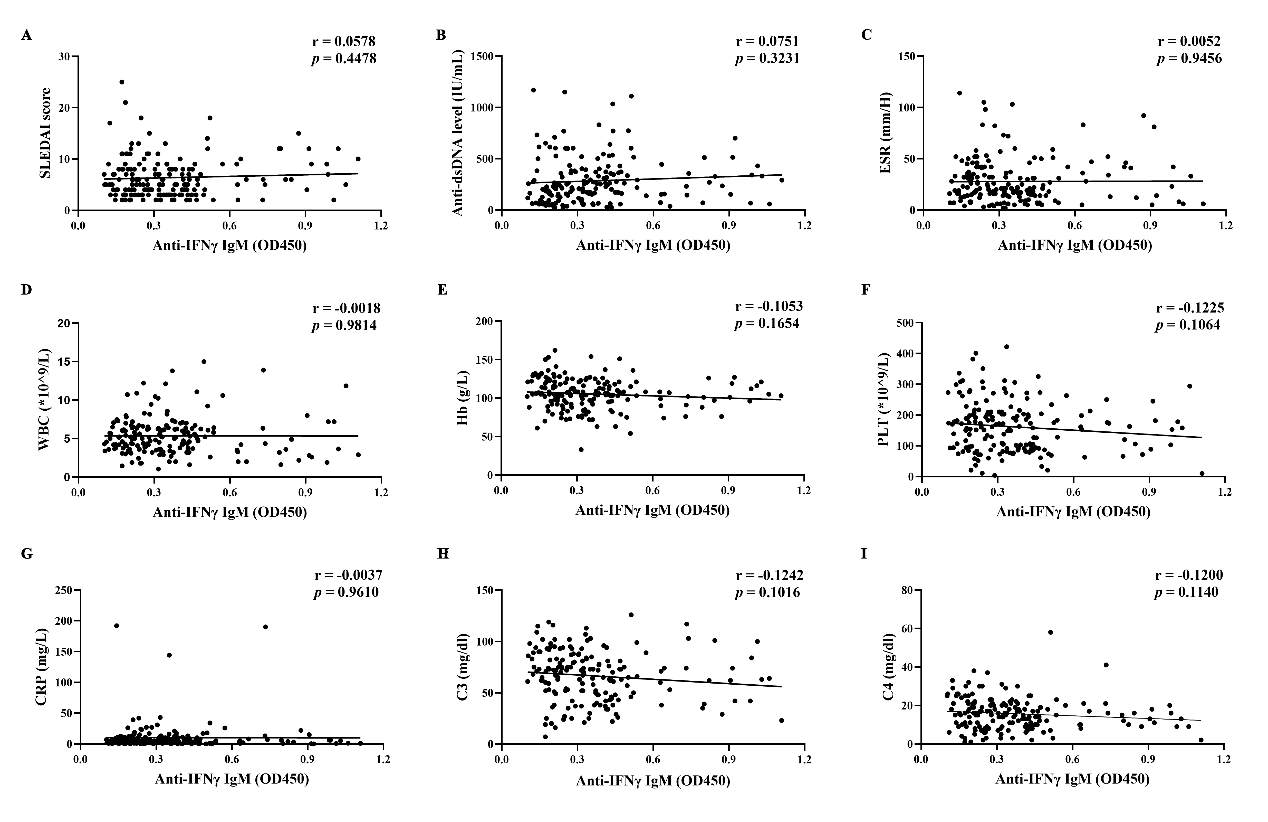


**Figure S4. The correlation of anti-IFN-γ IgM levels and laboratory data in patients with SLE.** The correlation between anti-IFN-γ IgM levels and SLEDAI score (A), anti-dsDNA Ab (B), ESR (C), WBC (D), Hb (E), PLT (F), CRP (G), C3 (H), and C4 (I) in SLE patients (n = 175). No significant difference was found. *SLE*, systemic lupus erythematosus; *SLEDAI*, SLE disease activity index; *Anti-dsDNA Ab*, anti-double-stranded DNA antibody; *ESR,* erythrocyte sedimentation rate; *WBC*, white blood cells; *Hb*, haemoglobin; *PLT*, platelets; *CRP*, C-reactive protein; *C3*, complement 3; *C4*, complement 4.


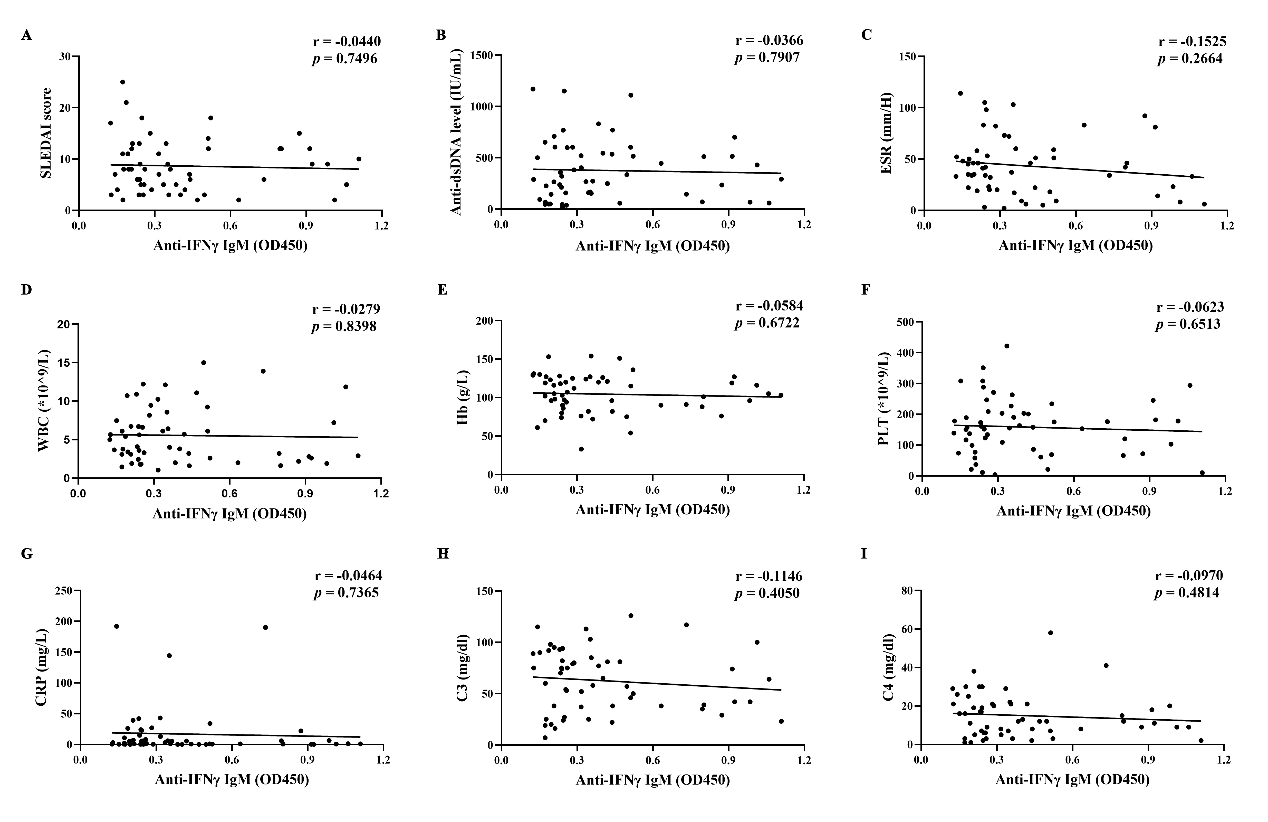


**Figure S5. The correlation of anti-IFN-γ IgM levels and laboratory data in SLE patients with severe infections.** The correlation between anti-IFN-γ IgM levels and SLEDAI score (A), anti-dsDNA Ab (B), ESR (C), WBC (D), Hb (E), PLT (F), CRP (G), C3 (H), and C4 (I) in SLE patients with severe infections (n = 55). No significant difference was found. *SLE*, systemic lupus erythematosus; *SLEDAI*, SLE disease activity index; *Anti-dsDNA Ab*, anti-double-stranded DNA antibody; *ESR,* erythrocyte sedimentation rate; *WBC*, white blood cells; *Hb*, haemoglobin; *PLT*, platelets; *CRP*, C-reactive protein; *C3*, complement 3; *C4*, complement 4.


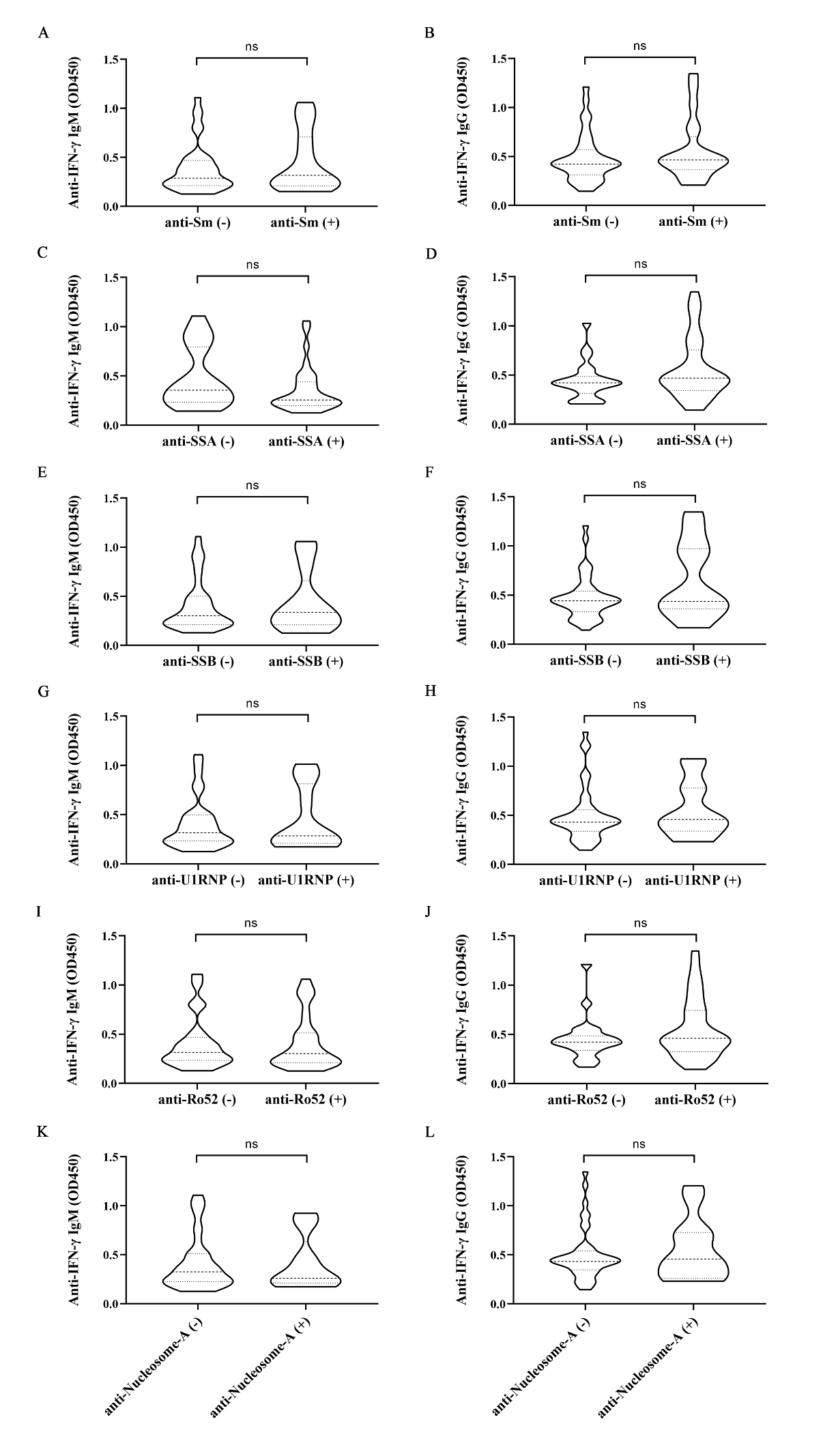


**Figure S6.** **The correlation of anti-IFN-γ IgG/IgM levels between patients with seropositive and seronegative autoantibodies.** The correlation analysis of serum anti-IFN-γ IgG (B, D, F, H, J, and L)/IgM (A, C, E, G, I, and K) levels between patients with seropositive and seronegative autoantibodies including anti-Sm (A and B), anti-SSA (C and D), anti-SSB (E and F), anti-U1RNP (G and H), anti-Ro52 (I and J), and anti-nucleosome-A (K and L) in SLE patients with severe infections (n = 55). No significant difference was found.

**
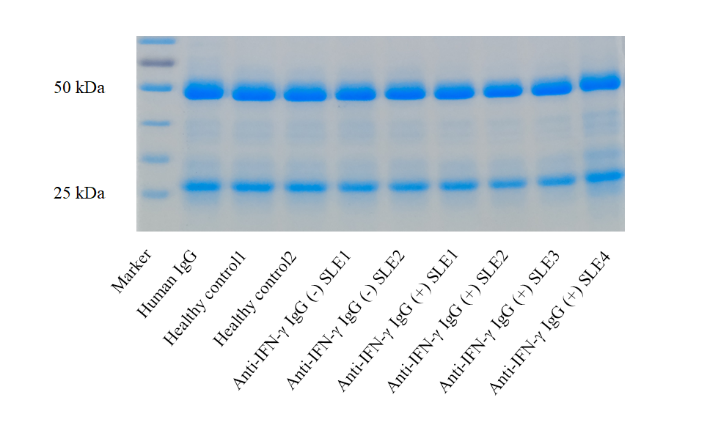
**

**Figure S7.** **Coomassie Brilliant Blue staining of purified total IgG from SLE patients.** The heavy chain and light chain of IgG were approximately 50 kDa and 25 kDa, respectively. The quantity of human IgG was 20 μg. The quantity of total IgG was 20 μg. *Human IgG*, unconjugated human IgG. *SLE*: systemic lupus erythematosus.


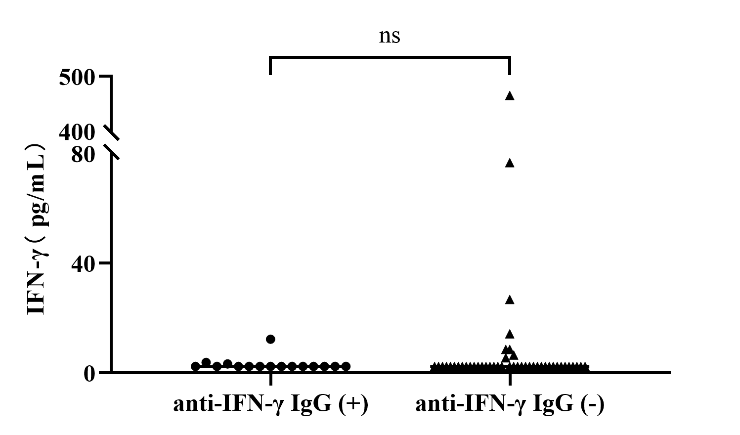


**Figure S8. Serum IFN-γ levels of SLE patients with severe infections.** The reference value was less than 23.1pg/ml, while the lower limit of detection was 2.4 pg/ml. No significant difference was found. *SLE*, systemic lupus erythematosus.
