# Supplementary material for: Neutralizing anti-IFN-γ IgG was increased in patients with systemic lupus erythematosus and associated with susceptibility to infection
Source: Clin Rheumatol. 2023 Oct 19;43(1):189–98. doi: 10.1007/s10067-023-06758-7 (PMC10774216; doi:10.1007/s10067-023-06758-7)

**Original WB image:**

**The first time:**

pSTAT1:


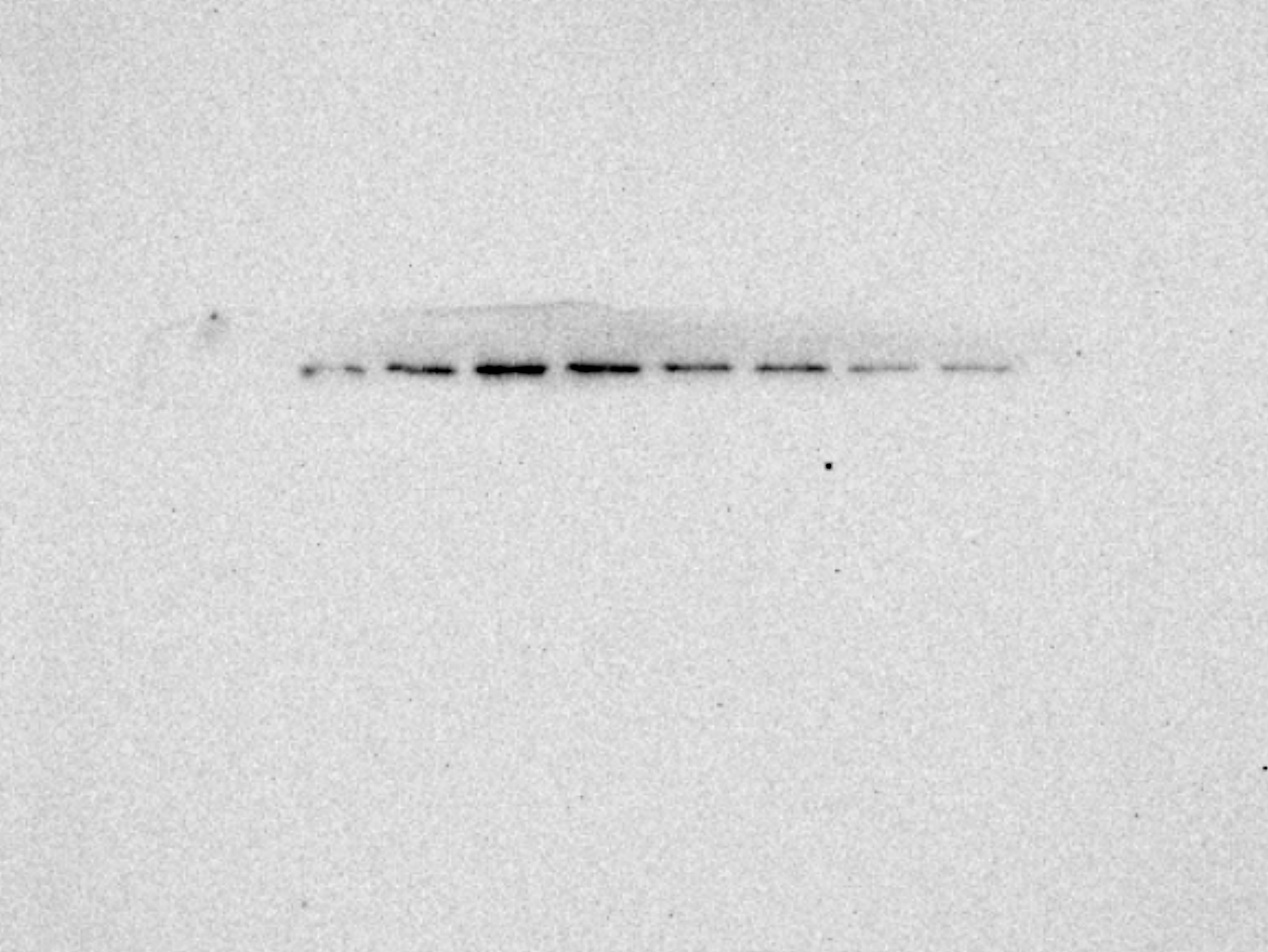


STAT1


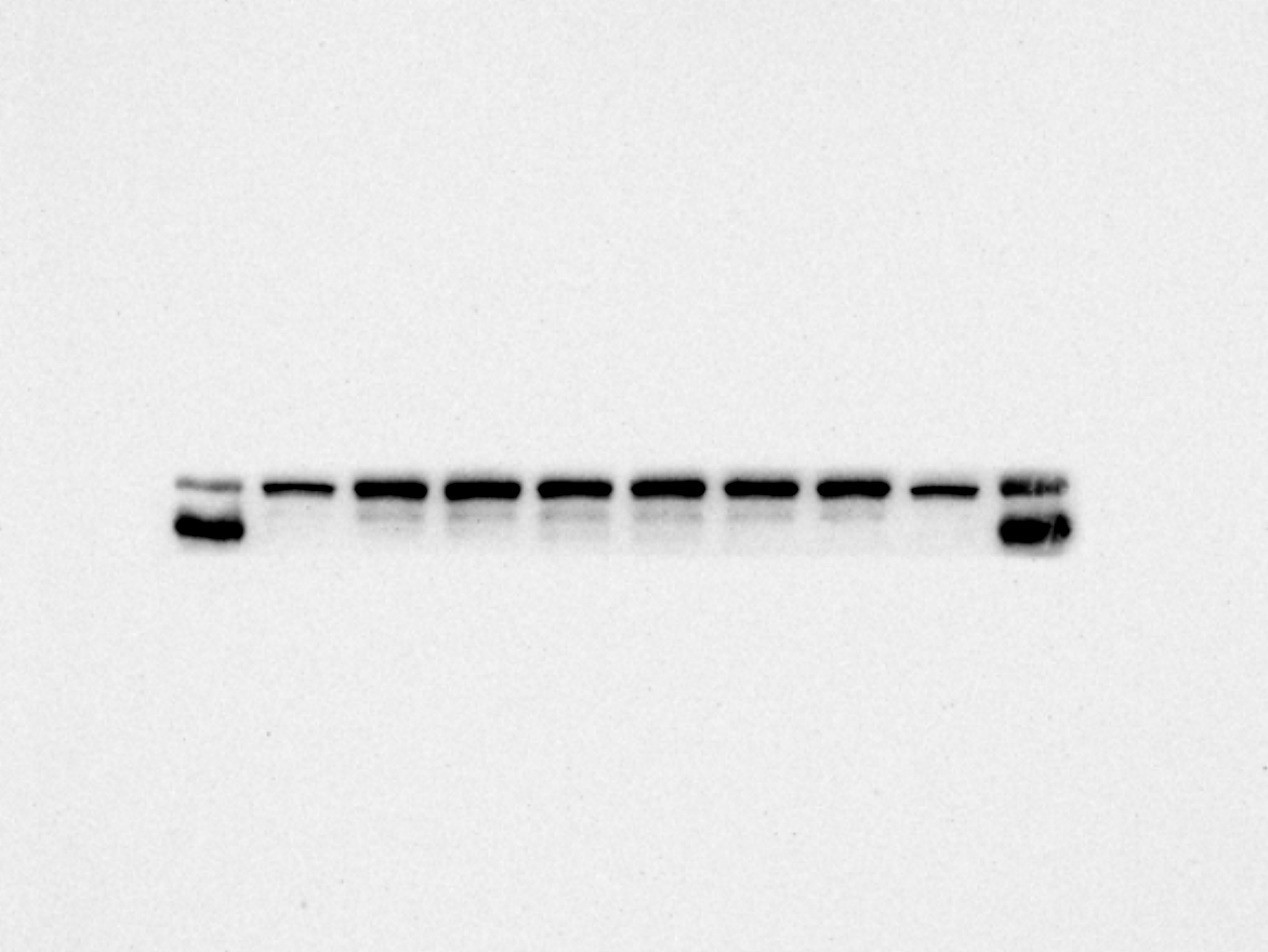


GAPDH:


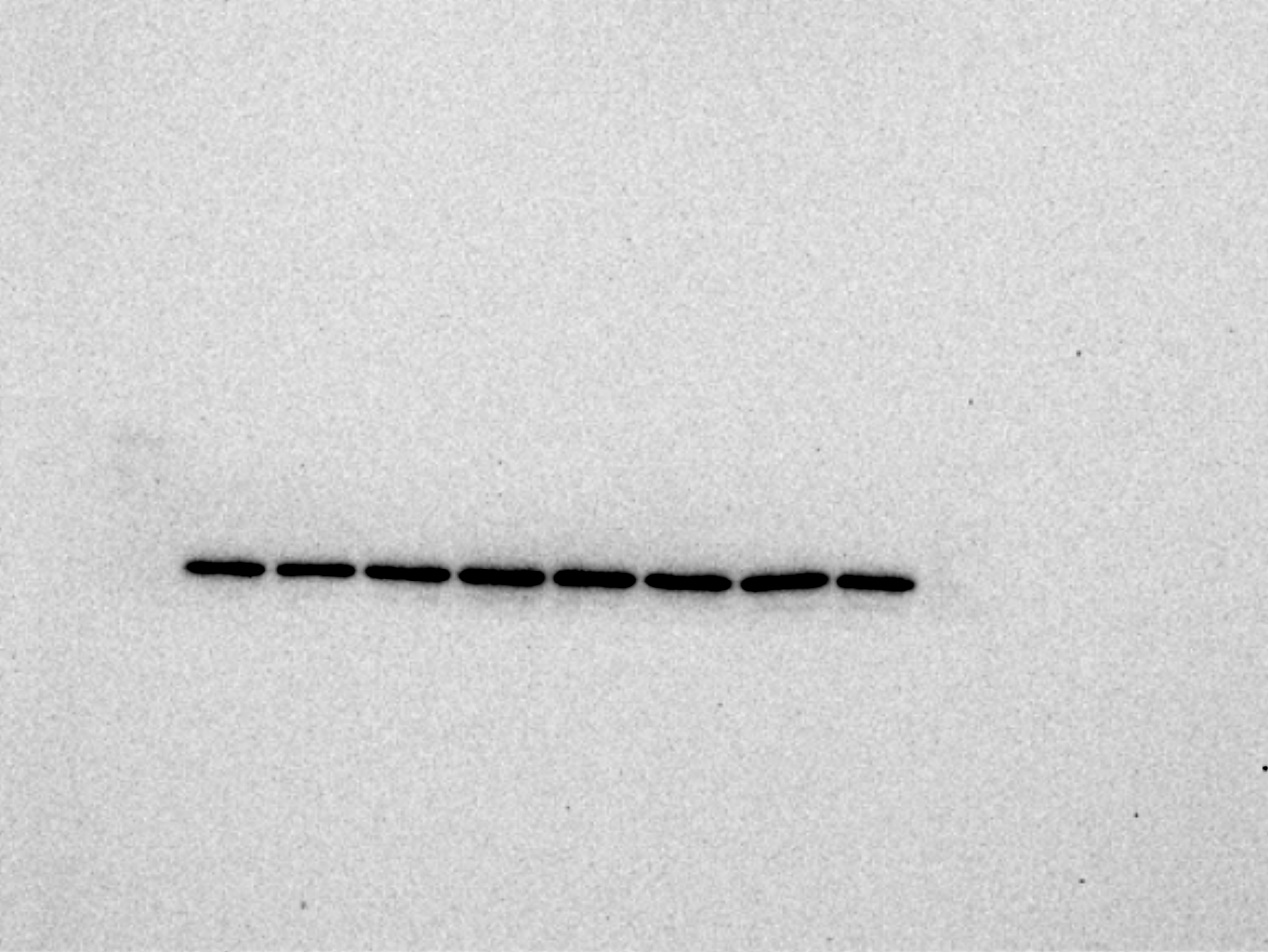


**The second time:**

pSTAT1


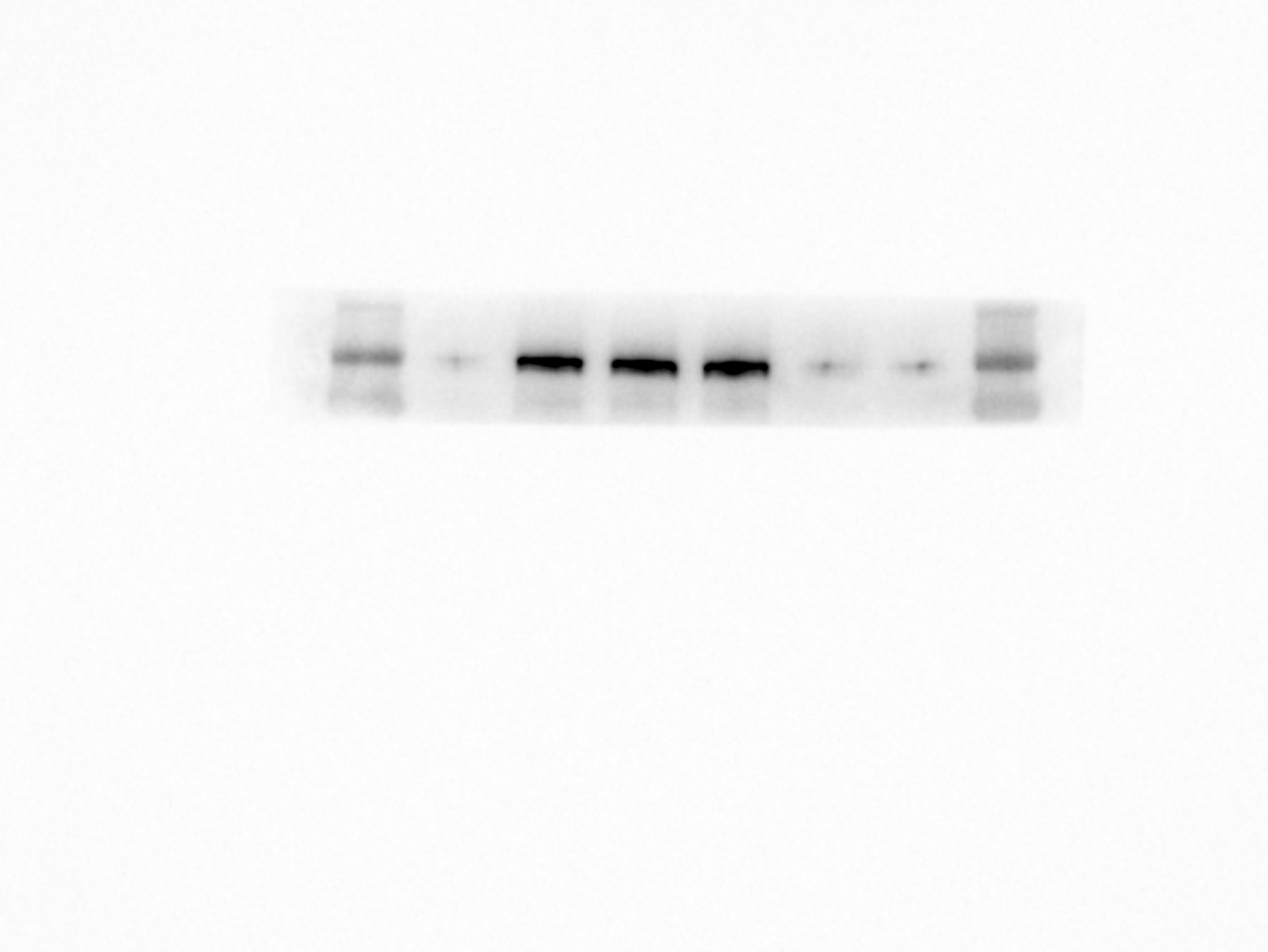


GAPDH:


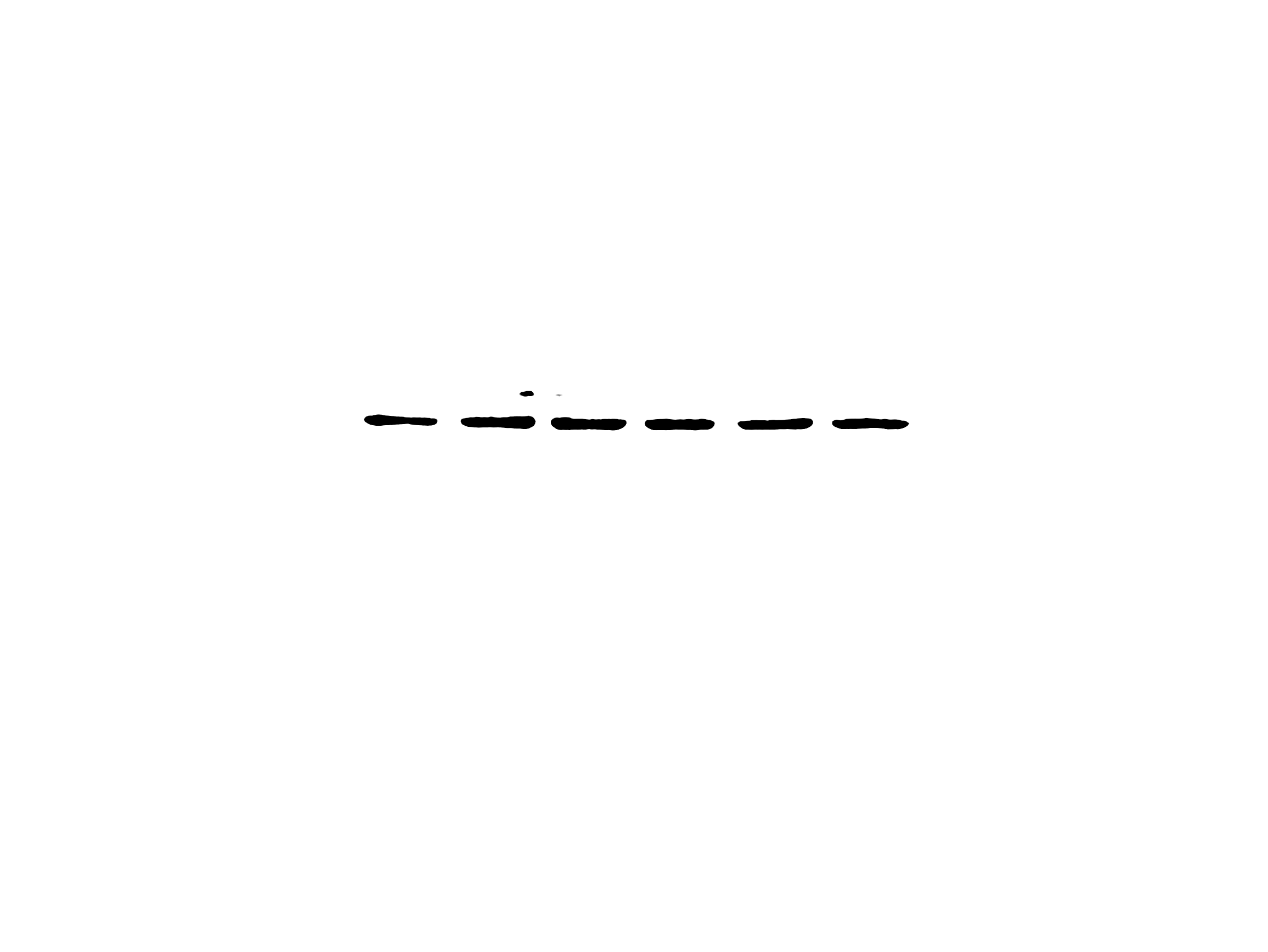


STAT1


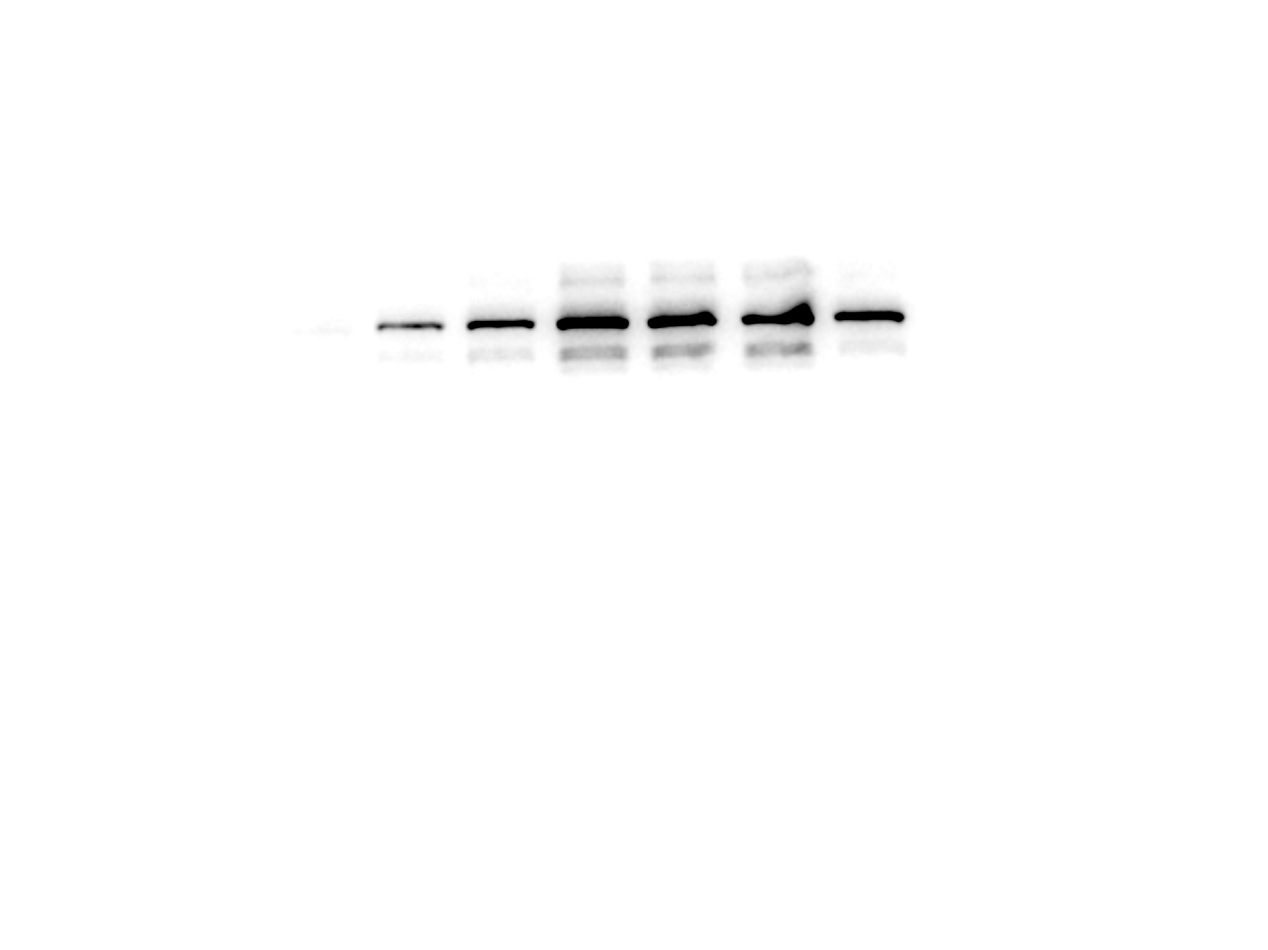


GAPDH:


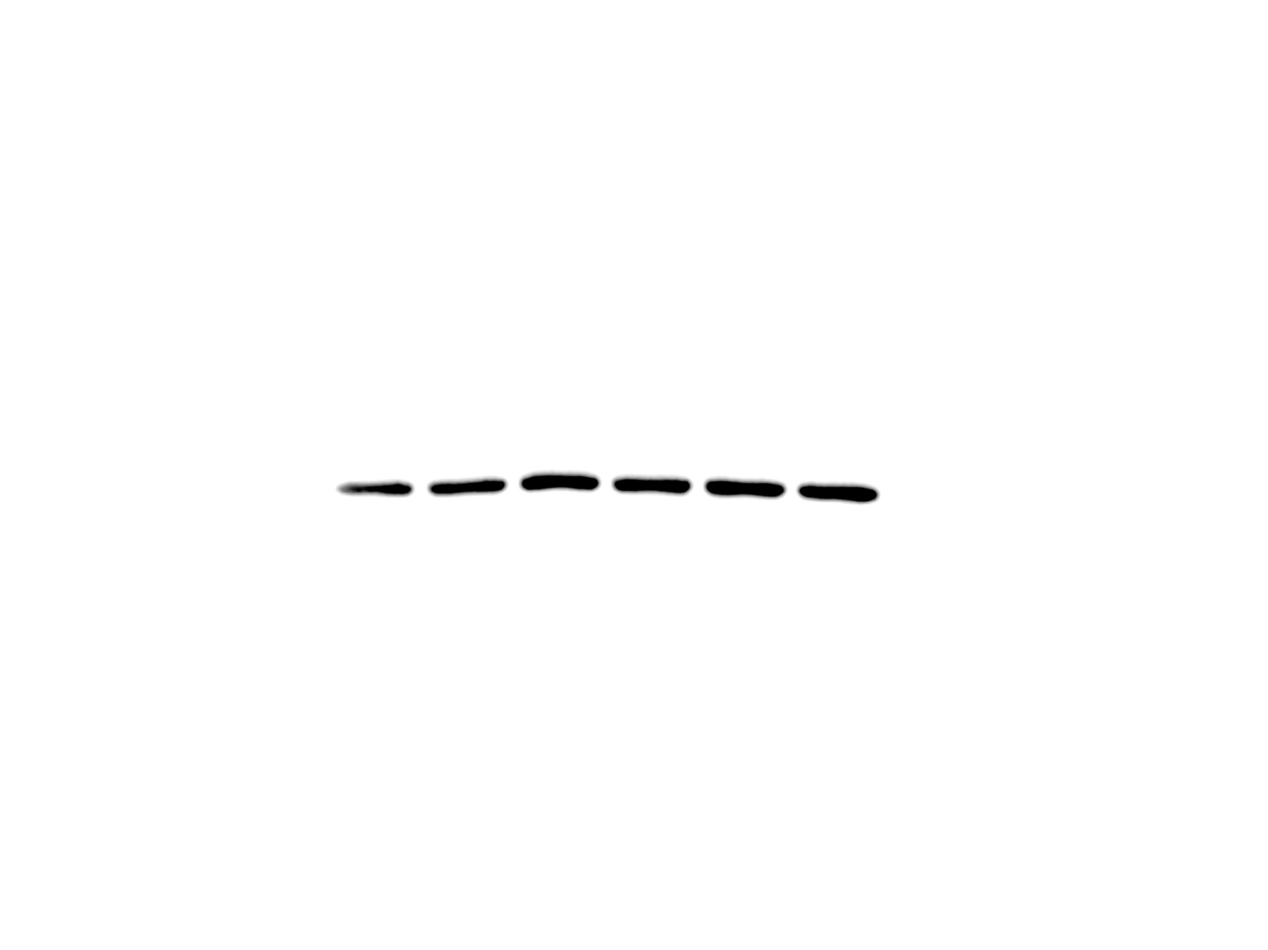


**The third time:**

pSTAT1


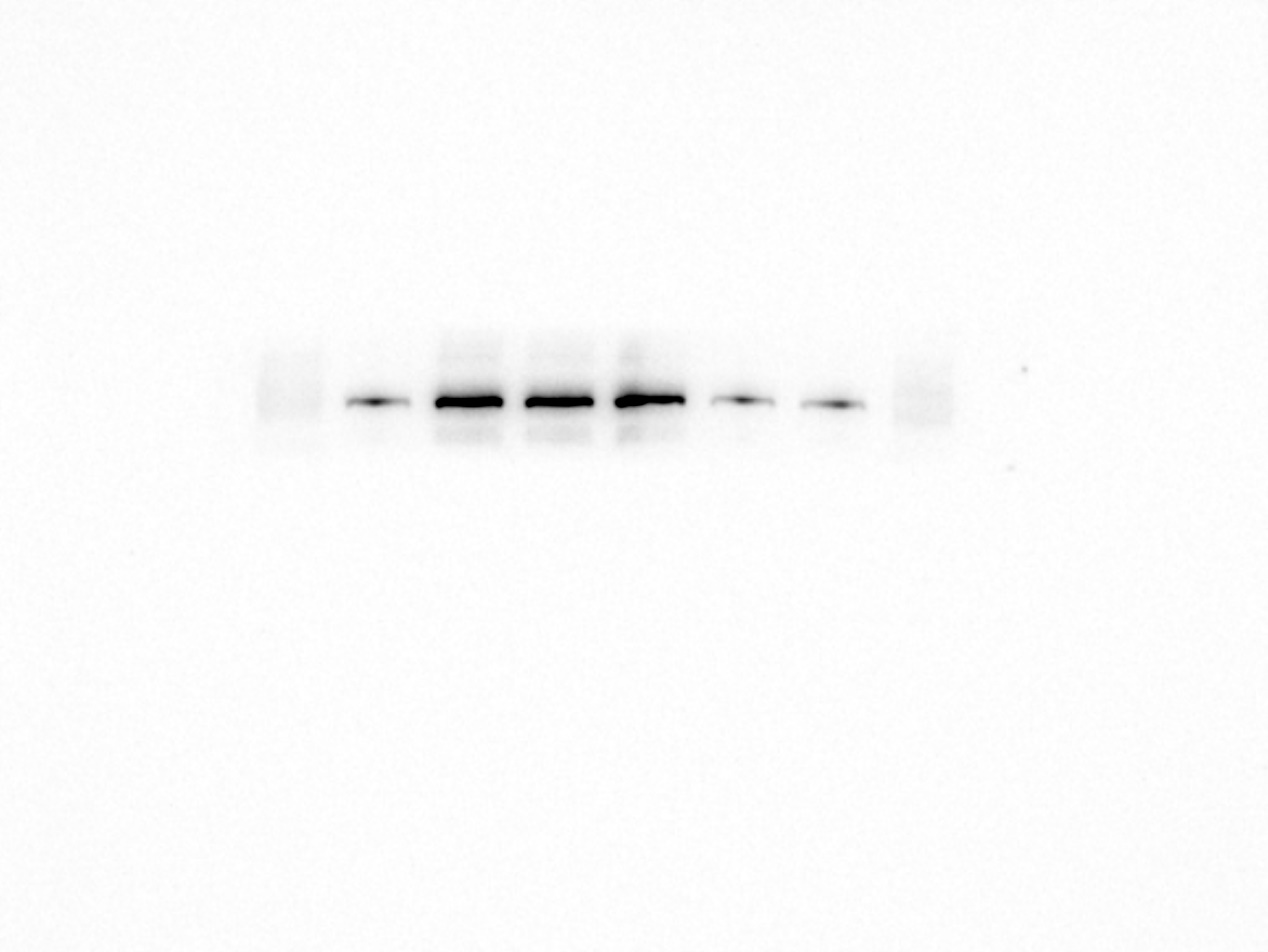


GAPDH


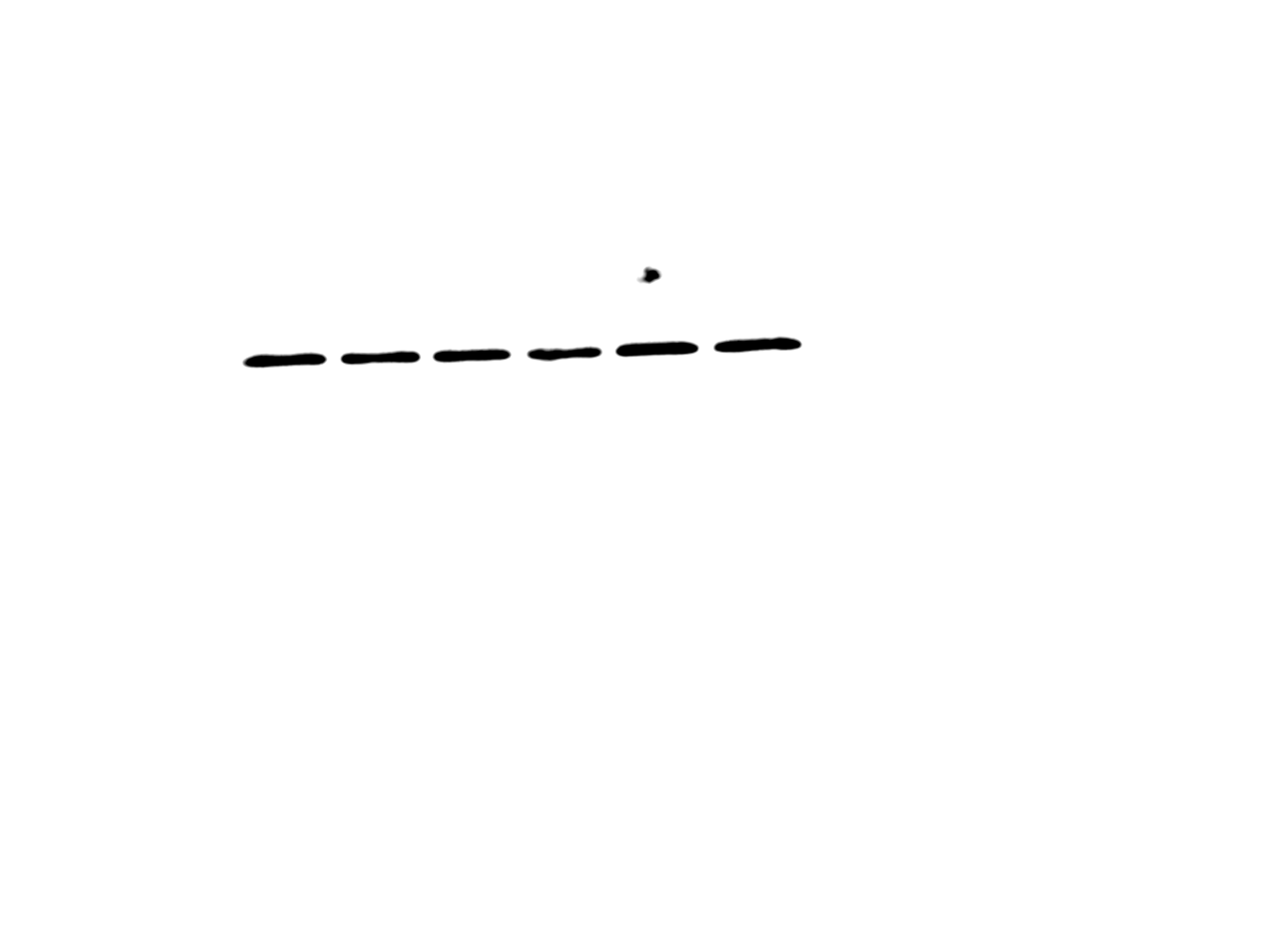


STAT1


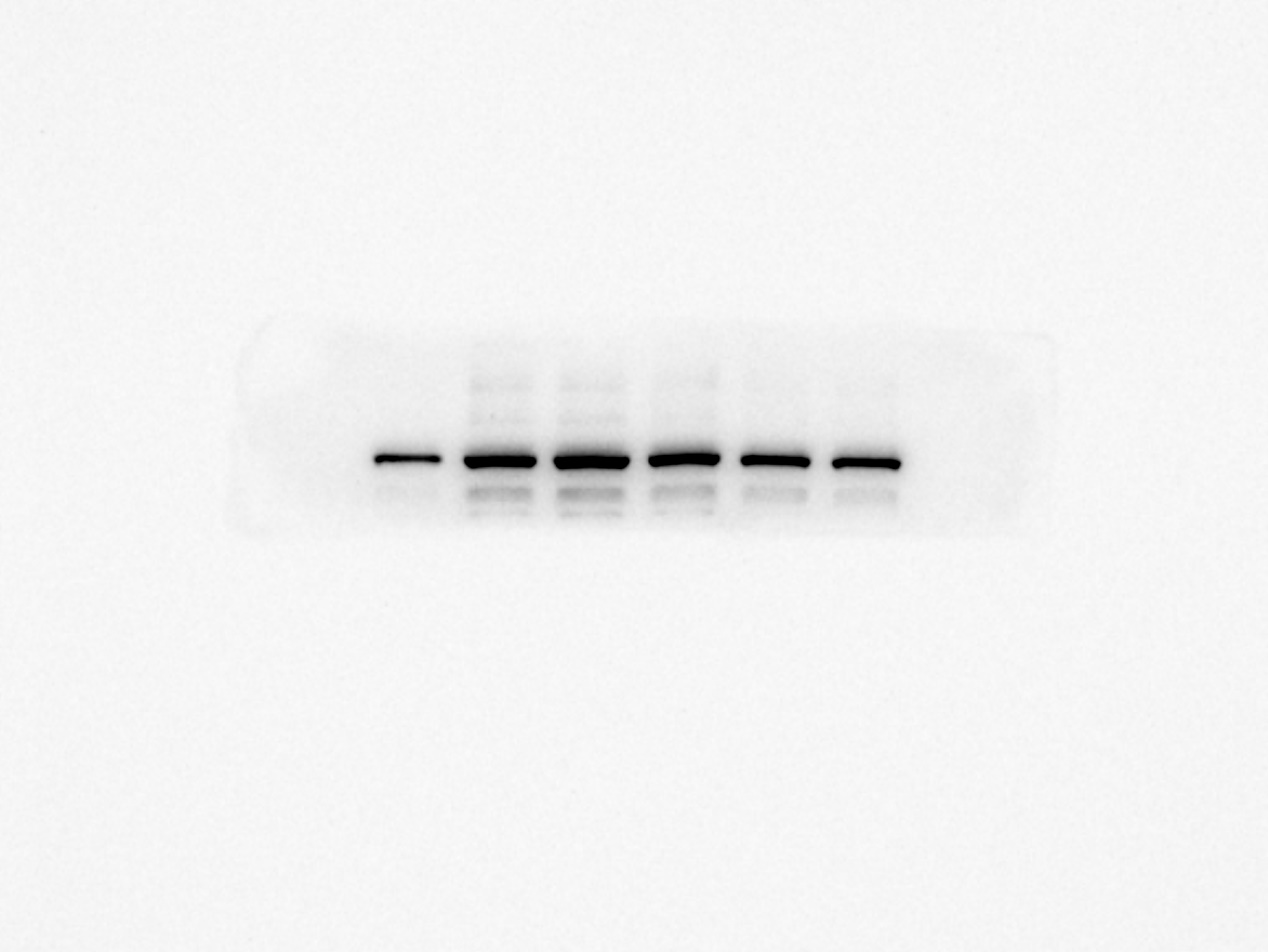


GAPDH:


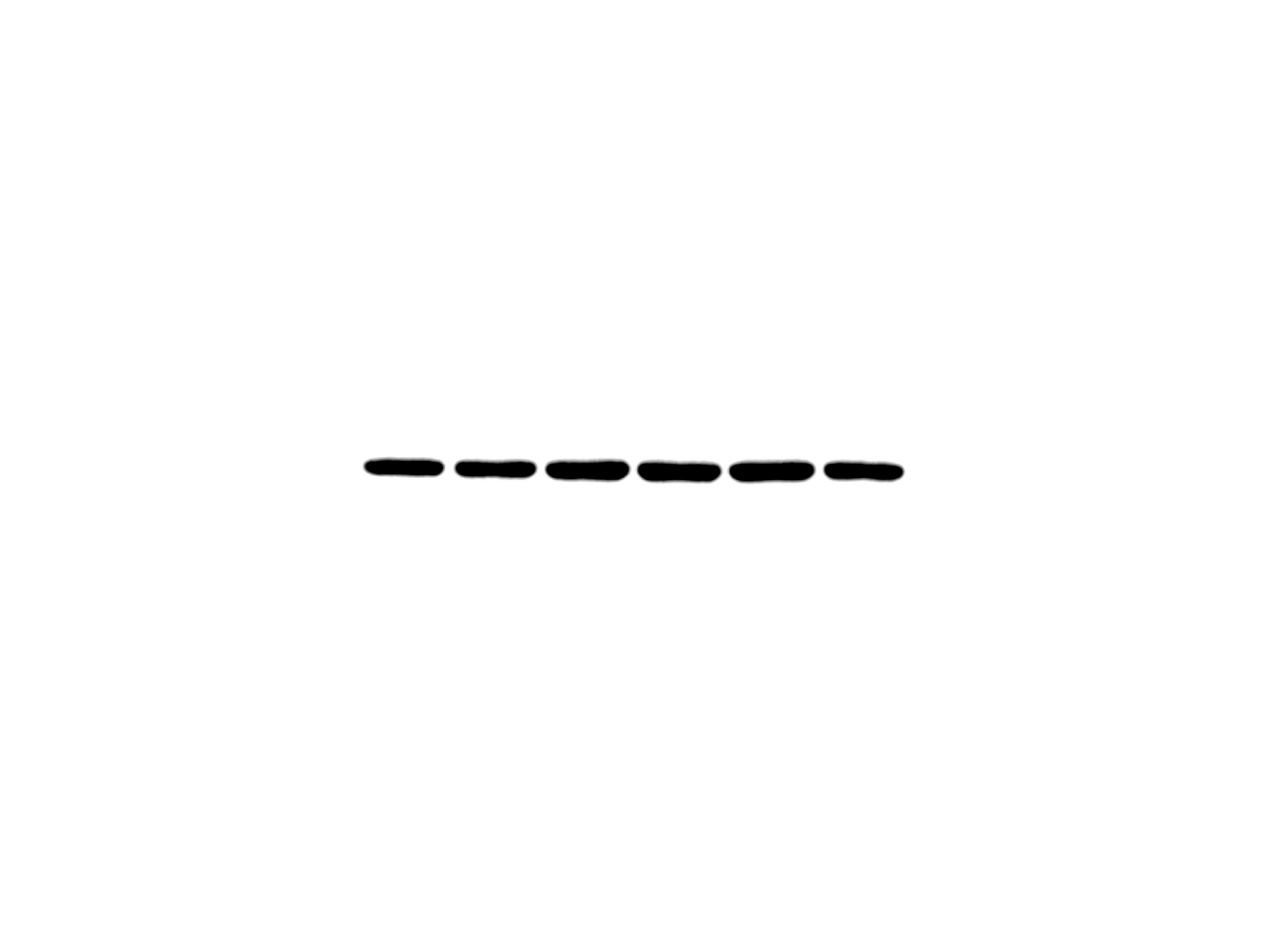

Supplement: Supplementary file 3 — Supplementary file3 (DOCX 1359 KB) [file 10067_2023_6758_MOESM3_ESM.docx]
